# Testing Education and Accountability as Moderators of the Self-Interest Bias in Moral Character Judgments

**DOI:** 10.1177/01461672251364308

**Published:** 2025-08-28

**Authors:** Yong Lu, Katarzyna Miazek, Konrad Bocian

**Affiliations:** 1 Department of Psychology in Sopot, SWPS University, Sopot, Poland

**Keywords:** self-interest bias, moral character judgments, motivated cognition, education, accountability

## Abstract

The self-interest bias describes an inclination of people to evaluate transgressors morally better when the latter’s actions serve the former’s interests. Effective interventions to mitigate this type of self-serving bias remain underexplored. The current research investigates education and accountability as potential moderators of this bias. In preregistered Study 1 (*N* = 668), as predicted, educating participants about the self-interest bias eliminated its effect on their moral character judgments. However, across three preregistered accountability studies (*N* = 2,239), holding participants accountable for their judgments did not attenuate the bias. Similarly, in preregistered Study 5 (*N* = 641), we found no evidence of a combined effect of education and accountability on reducing the bias. These findings suggest that while increasing awareness through education may effectively reduce biased moral judgments, accountability, especially when lacking external scrutiny or consequences, does not. Theoretical implications, methodological limitations, and future research opportunities are discussed.

Each of us is central to our universe. Rational theories assume that judgments are consistent and stable. However, social concepts such as self-interest (i.e., egocentricity) and norms (e.g., justice) can be relevant to important judgments and yet support conflicting preferences. These influences can give rise to systematically biased judgments rooted in egocentric perspectives. One such well-documented egocentric bias relates to how self-interest influences moral judgments, henceforth referred to as the self-interest bias. This bias occurs when individuals tend to make more lenient moral judgments of transgressors whose immoral actions serve their own interests, compared to when those actions do not (Bocian et al., 2016; Bocian & Wojciszke, 2014a). Much of the evidence suggests that the self-interest bias is largely mediated by people’s liking toward agents (Bocian et al., 2018, 2022; Bocian, Baryla, et al., 2022) and operates out of awareness (Bocian & Wojciszke, 2014b), as indicated by an inclination to trust those who cheat experimenters (Bocian et al., 2016).

Furthermore, the self-interest bias is observed on a group level (Bocian et al., 2021), toward hypocritical transgressors (Bocian et al., 2024), among preschool children (Myslinska Szarek et al., 2021), with the attribution of moral responsibilities to AI (Dong & Bocian, 2024), and in fairness decision-making of AI agents (Miazek & Bocian, 2025). Given that this implicit bias skews moral perceptions of others, which are fundamental for impression building and trust (Brambilla et al., 2021), it is crucial to explore how to mitigate this bias. In what follows, we first briefly propose theoretical explanations of the self-interest bias, followed by a series of studies examining education and accountability as potentially debiasing interventions in combating the self-interest bias.

## Theoretical Explanations of the Self-Interest Bias

Like many cognitive biases in human decision-making and judgments, the self-interest bias can be explained by dual-process models in terms of a complex interplay of two qualitatively different thinking systems—Type 1 and Type 2 (Tversky & Kahneman, 1986). According to this view, Type 1 processing is fast, implicit (automatic), intuitive, and sometimes associated with affect. By contrast, Type 2 processing is slow, explicit (controlled), rational, and somewhat more voluntary than Type 1 processing. The two types of processing can be mapped onto two different arms of moral thoughts. By and large, Type 1 processing is associated with concerns about the violation of deontological rules (e.g., the rules of self-interest), whereas Type 2 processing relates to utilitarian considerations of maximizing expected welfare (Greene & Haidt, 2002). Emotion-laden moral judgments often resemble instant perceptions rather than deliberate inferences (Haidt, 2008); that is, moral cognition is mainly generated by Type 1 rather than Type 2 processing. Since the self-interest bias is primarily bound to self-anchored, egocentric evaluations (Bocian et al., 2020), we argue that the bias is most probably generated by Type 1 processing (Bocian et al., 2016).

The self-interest bias can also be explained by fuzzy-trace theory, a dual-process model of memory, judgments, and their development (Brainerd et al., 2015). The theory posits that individuals encode in parallel two types of memories: gist (intuitive) traces that store the semantic and relational content of experienced events or objects, and verbatim (analytical) traces that contain the details of particular targets. Moreover, gist-based processing is qualitative, vague, and impressionistic and operates on the essential bottom line of judgment information, whereas verbatim-based processing is quantitative, vivid, and precise. Importantly, it seems that when making moral judgments, individuals whose self-interest is involved rely more on a gist level of processing because self-interest is general and categorical. By contrast, individuals whose self-interest is noninvolved or even harmed rely more on a verbatim level of processing because they need to distinguish the underlying details of (im)moral behaviors or even calculate their cost (Reyna & Brainerd, 2011).

Further support for this premise comes from an empirical study on the retrospective recall of others’ cheating and the personal consequences of cheating to the self (Bell et al., 2014). The authors showed that observers remember cheaters very well when the cheaters’ immoral behaviors are associated with the observer’ personal *costs*, as represented by verbatim processing. However, it is not true when these behaviors are associated with the observers’ *benefits*, as represented by gist processing. In sum, the self-interest bias can be considered as a type of memory distortion when individuals tend to eschew verbatim representations of exact details of and the relevant gist of others’ immoral behavior and to use simplified gist information instead.

## Education and Accountability as Potential Moderators of the Self-Interest Bias

Educating people on how to prevent biases alters their decision-making and judgments by the cued norm (e.g., Matute et al., 2015; van Brussel et al., 2021). That is, people who are dispositionally or temporarily activated in normative considerations are motivated to be most likely to behave in norm-consistent ways. For example, people show small-scale but reliable bias reductions when asked to avoid potential bias in a single social category (Axt et al., 2019). Educating preclinical medical students about cognitive biases leads to better clinical decision-making than practicing physicians (Hershberger et al., 1996). As an educational intervention, a brief forewarning is observed to be effective in mitigating judgment biases, such as anchoring effects (Epley & Gilovich, 2005) and the hindsight bias (Nestler & Egloff, 2009), and in ignoring invalid information (Schul, 1993). Given the strong evidence that moral judgment development is related to education, research interests have shifted to the exploration of which educational contexts promote growth (e.g., Maeda et al., 2009).

Bocian, Baryla, et al. (2022) provided evidence that educating people that attitudes bias moral character judgments can ameliorate the liking bias—a bias engendered by those who judge agents morally better (worse) when they know that their attitudes are (dis)similar to the agents. Building on this prior study, the current research aimed to investigate whether educating individuals about the clear-cut descriptive norms, specifically, that egoistic motivations can bias moral character judgments (e.g., that judgments should be based not on self-interest, but rather on considerations of justice and broader social and ethical standards) could attenuate the self-interest bias. We thus predicted that the presence or absence of educational instructions on the self-interest bias would moderate moral character judgments. Thus, we propose the following hypothesis:

**Hypothesis 1.** The self-interest bias in moral character judgments will be reduced or eliminated under *education* conditions compared to *non-education* conditions.

Accountability pressures feature a ubiquitous rule in human social systems: in essence, one must justify his or her judgments to others. When faced with the necessity to generate persuasive arguments for their judgments, people may switch to more effortful and self-critical information processing (Lerner & Tetlock, 1999). A fMRI study indicated that when held accountable, people show greater activation in mOFC, a region implicated in reducing the self-serving biases (Hughes & Beer, 2012). This region is a critical component of a neural circuit mediating effort-related responding (Münster et al., 2020).

Specifically, holding people accountable for their judgment *processes*, a focus on the course of inputs by which the judgment was made, and to their judgment *outcomes*, a focus on outputs, regardless of the judgment course, are likely to motivate them to proceed with information in more vigilant and complex ways (Frink & Klimoski, 1998). As a result, it has often been noted that accountability helps dampen a broad array of inferential biases (e.g., Novicevic et al., 2008; Simonson & Nye, 1992). Evidence has further shown that process accountability, relative to outcome accountability, consistently helps improve judgment quality in a variety of tasks (e.g., Verwaeren et al., 2015; but see Chang et al., 2017 for failures). Besides, these two accountability systems function incongruently in resource distribution, such that process accountability subsumed under equality norms leads to excessive conformity to these norms; whereas, outcome accountability subsumed under equity norms leads to deviation from these norms (Patil et al., 2017).

Again, Bocian, Baryla, et al. (2022) found that holding participants accountable for their judgment processes, as informed at the outset of the study that a researcher would later interview them to understand what type of information they used to form impressions of agents, eliminated the liking bias. Other researchers also championed this concept as efficient in accounting for moral judgments (e.g., Bersoff & Miller, 1993). Therefore, we aimed to investigate whether process and outcome accountability moderate the self-interest bias in moral character judgments. We propose the following hypothesis:

**Hypothesis 2**. The self-interest bias in moral character judgments will be reduced or eliminated under *accountable* conditions (both process and outcome) compared to *unaccountable* conditions.

## The Present Research

**Figure 1.**
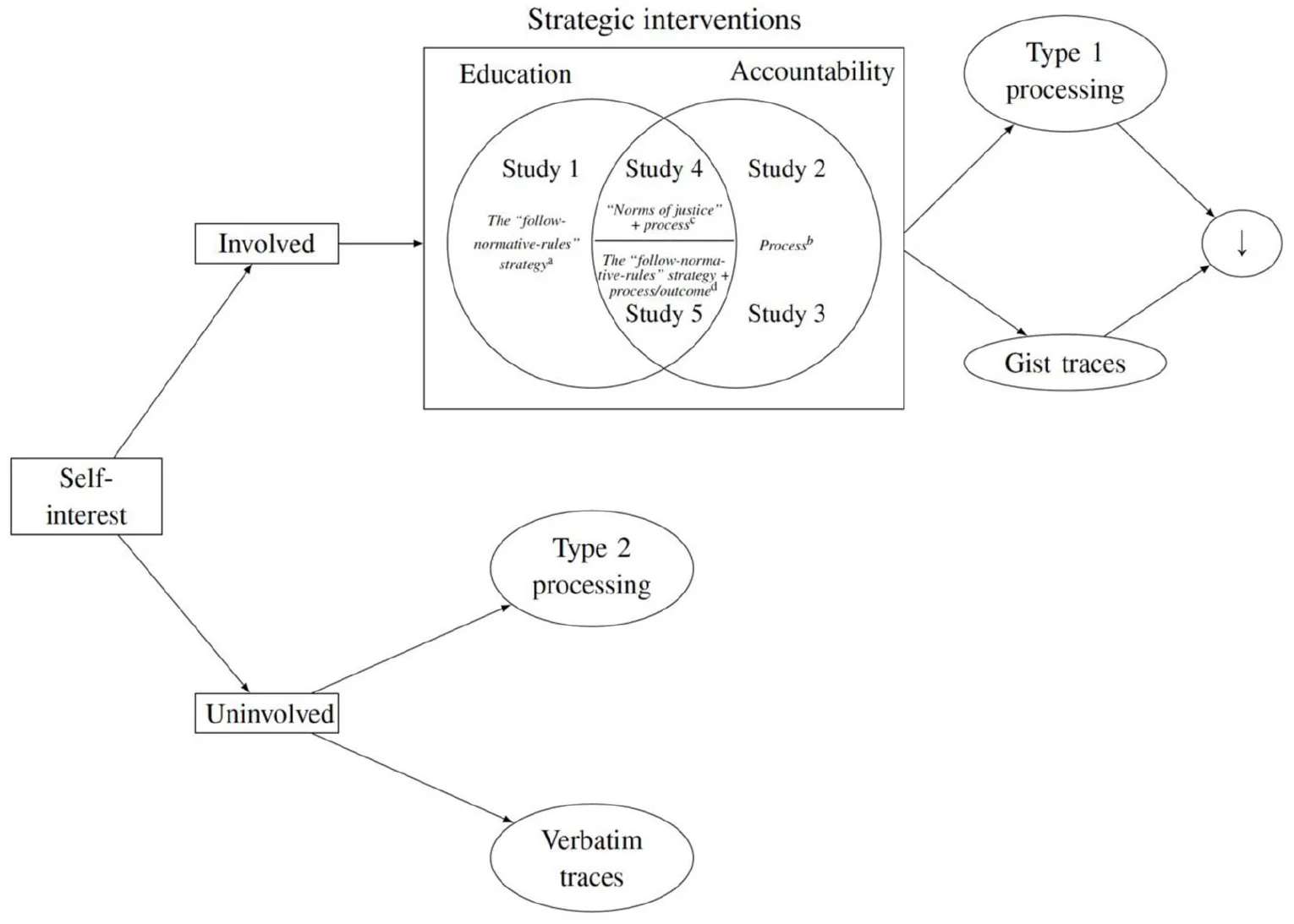
Education and accountability as potentially strategic interventions to lessen the self-interest bias: Conceptual framework, underlying mechanisms, and the scope of the present research. *Note*. ^a^In Study 1, *the “follow-normative-rule” strategy* indicates educational instructions on the concept of the self-interest bias in moral character judgments and on how to mitigate this bias. ^b^In Studies 2 and 3, the *process* indicates a strategic intervention of process accountability, in which participants were asked to justify the reasons for their moral character judgments. ^c^In Study 4, the *“norms of justice” + process* indicates a jointly strategic intervention of education and accountability, in which participants were asked to justify the reasons (process accountability) for their moral character judgments according to social and ethical standards such as norms of justice (education). ^d^In Study 5, *the “follow-normative-rule” strategy* + *process/outcome* constitutes a joint intervention targeting both cognitive and motivational mechanisms. In these conditions, participants received instructions on applying the “follow-normative-rule” strategy (education) and were additionally asked to either (a) justify how they reached their moral judgments by describing their reasoning and decision-making process (*process* accountability), or (b) justify the accuracy or effectiveness of their moral judgments (*outcome* accountability). *Key*: ↓ = dampening.

We conducted five studies testing education and/or accountability as potential moderators of the self-interest bias. In doing so, it also enables us to type on the boundary conditions of the occurrence of this bias when education and accountability, represented as directly instructional and indirectly motivated reasoning interventions, respectively, are introduced. Specifically, Study 1 examined whether educating people that self-interest is prone to distort our moral character judgments could be an instructional tool to reduce the self-interest bias. Following a similar study setup to Study 1, Study 2 investigated the effect of process accountability on alleviating this bias, followed by Study 3 as a replication in a different and more rigorous study setup. Both Studies 4 and 5 tested the joint effects of education and accountability (process and outcome) on attenuating this bias (cf., Figure 1).

**Figure 2.**
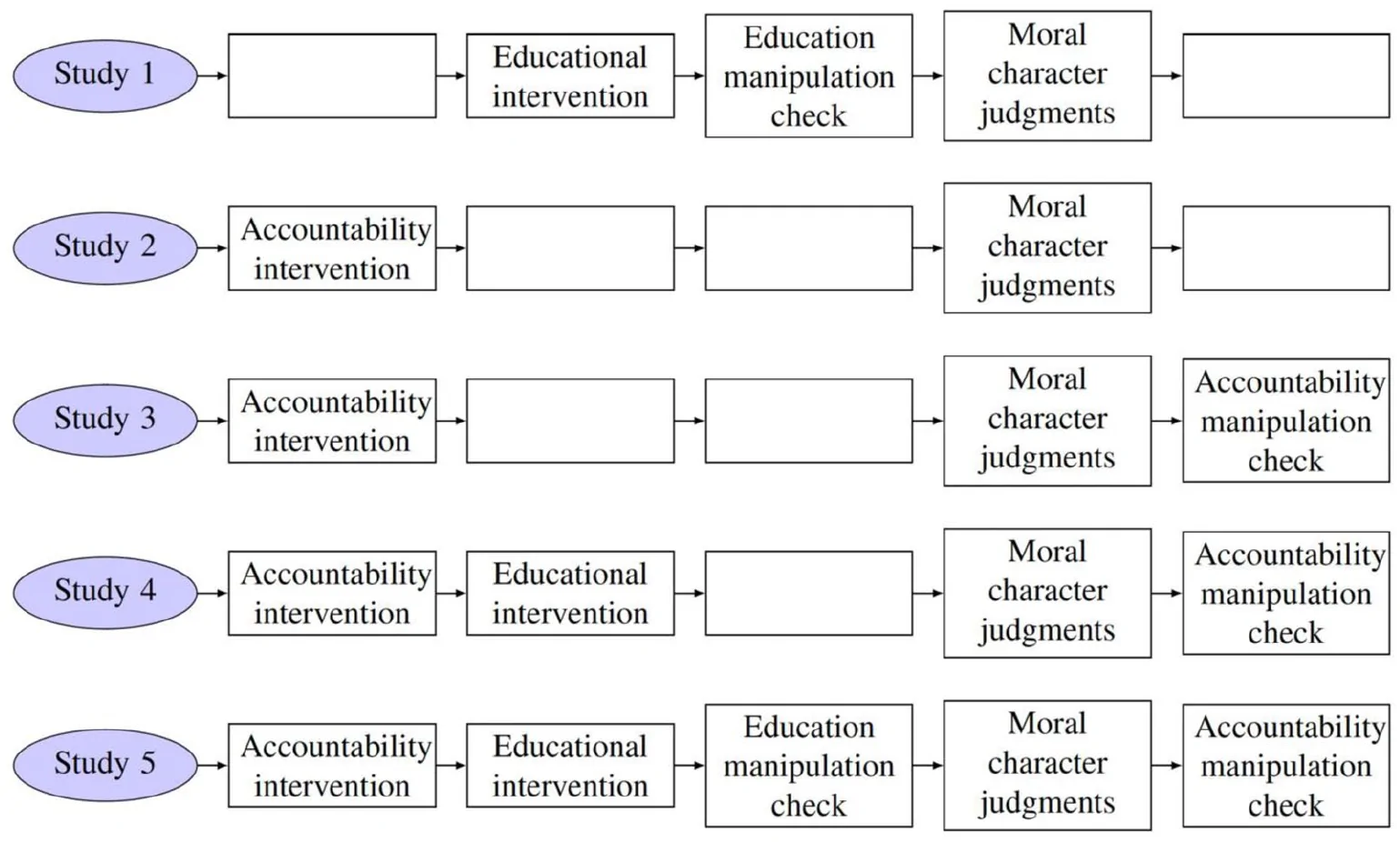
Schematic overview of the present research.

Across the present studies, approximately half of the participants were assigned to either an educational intervention (Studies 1 and 5) or one of several accountability manipulations (Studies 2 through 5), while the remaining participants served as controls—receiving neither education nor accountability prompts (cf., Figure 2). Specifically, we tested seven experimental or control conditions: (1) an education condition, (2) a process accountability condition, (3) an outcome accountability condition, (4) a rigorously joint education and accountability condition emphasizing norms of justice, (5) a non-education control condition, (6) an unaccountability control condition, and (7) a non-accountability control condition. Conditions 5 through 7 were designed to serve as parallel controls to conditions 1 through 4, allowing us to assess the moderating effects of education and various forms of accountability on the self-interest bias in moral character judgments.

While Studies 1 and 2 focused on breaches of distributive justice, Studies 3 through 5 employed a variety of moral vignettes. Across all studies, the central focus was on how individuals judge the moral character of transgressors—evaluations that involve considerations of justness, morality, reliability, honesty, trustworthiness, and commitment to moral values. These dimensions are fundamental to impression formation and play a central role throughout its stages, from initial assumptions to information gathering and final assessments (Brambilla et al., 2021). Thus, these moral character judgments are crucial to social perception and have a vital impact on trust, cooperation, and social acceptance (Crockett et al., 2021).

Following previous work (Bocian & Wojciszke, 2014a), we operationalized the self-interest bias as the difference in participants’ moral character judgments of agents whose actions were unjust or unethical but beneficial to the participants themselves versus agents whose actions, either just, unjust, or unethical, depending on the study, did not benefit the participants. In essence, when an agent’s unjust actions serve an evaluator’s self-interest, the resulting positive affect, namely, liking toward the agent—often occurring outside of conscious awareness—can lead to more pronounced, favorable moral evaluations of the agent. This pattern contrasts with evaluations of agents whose actions, regardless of moral justness or ethics, are not beneficial to an evaluator. All study pre-registrations, raw data files, analysis scripts, additional results for exploratory variables, and study materials—including stimuli and variables used in the Qualtrics surveys—are available for download from the Open Science Framework at https://osf.io/gnz7c.

## Study 1

Study 1 examined Hypothesis 1, which proposes that educating individuals about the self-interest bias can reduce or eliminate its influence on the moral character judgments. We expected that the self-interest bias would emerge in the non-education condition, such that participants would judge a decider who made an unjust decision benefiting themselves as more moral than one who made a just decision benefiting someone else. By contrast, in the education condition, we predict that this bias would be reduced or eliminated, such that participants would evaluate a decider who made an unjust decision as equally moral as one who made a just decision. The hypothesis, method, statistical analysis, and sample size determination were preregistered at https://aspredicted.org/h9c_bbq.

### Method

In this article, we report all measures and any data exclusions. Any additional measures not included in the primary analyses are reported in the Supplemental Materials. The relevant Research Ethics Committees have approved all studies. All participants provided informed consent and took part voluntarily. Participants did not participate in any of the other studies for each study. The samples of all participants were administered online using Qualtrics surveys linked through the Prolific Academic platform’s user interface. Priori power analyses were conducted using G*Power (Faul et al., 2007) for sample size estimations, based on effect size estimates from earlier conducted and similar experiments to achieve at least a standard sensitivity of the hypothesis testing procedure (i.e., power) of 95% under a fixed α level of 5%, before exclusions. In this article, the conclusions of significance tests were generally not dependent on the exclusion criteria, with analyses repeated on the entire sample leading to similar results in all studies (see the Supplemental Materials).

#### Participants

Based on a pilot study’s estimated medium interaction effect size of *f* = 0.14, a total sample size of 665 was required to achieve sufficient power to replicate this effect. To allow for potential dropouts, we recruited 784 participants from the United States, of which 96 were excluded for non-responding to the survey questions (*n* = 59), a low level of engagement in the task (*n* = 34), or incorrect answers to an obvious attention check question (i.e., pick the number six; *n* = 3). Excluding these invalid and non-responding participants, the rest of our exclusion percentages (i.e., 5.1%) were within the range of published exclusions for data collected on Prolific (e.g., Geldsetzer, 2020; Meeussen & van Laar, 2018; Stanton et al., 2022). Thus, the final sample consisted of *N* = 688 participants (*M*_age_ = 40.0 years, *SD* = 13.1; 48.7% female; 48.8% male; 2.5% unspecified) for inclusion in this study. For ethnic categories, see the Supplemental Materials.

#### Design

The study employed a 2 (Decision: Unjust but Self-Beneficial vs. Just but Other-Beneficial) × 2 (Education: Yes vs. No) between-subjects design. In the *unjust but self-beneficial* condition, the agent made a biased decision that financially benefited participants. By contrast, the *just but other-beneficial* condition involved the agent making a fair decision that favored a third party at a cost to participants, thereby prompting them to weigh their self-interest against fairness. Furthermore, half of the participants received an educational intervention describing the concept of the self-interest bias in moral character judgments and instructing them on how to counteract this bias using a “follow-normative-rules” strategy (see the Method and Materials Supplement for exact wording). The other half received no such information. The education manipulation was designed to shift participants’ focus from self-interest to impartial justice concerns, thereby impeding susceptibility to the self-interest bias.

Following conventions in the literature (e.g., Cialdini et al., 1990), and to ensure that participants in the education condition understood and accepted the core message of the intervention, we asked them whether the following two statements were true or false after reading the instructional material: (a) “The self-interest bias is a tendency to judge people as more moral when their action benefits us, albeit violates a norm, such as fairness, and less moral if the same action benefits someone else.”; and (b) “To avoid the self-interest bias, our judgments on people should depend on whether their actions are against any norm, such as fairness, rather than [sic. on] our personal interests.” These two questions served both to verify that participants had read and comprehended the educational content and to reinforce the manipulation by prompting active engagement with its core principles.

#### Procedure

At the outset, participants were informed that they would receive a base payment of £0.90, along with a potential bonus, for their time spent in the study. They were then told that they would be paired with a “decider” and another participant, and that this decider would later determine how much of a £1 bonus they and the other participant would lose based on their performance in a subsequent main task. In reality, both the decider and the other participant were fictitious and controlled by the experimenter. To enhance the believability of this setup, participants were shown a simulated environment where other individuals appeared to be joining the study. Within a mock chat room, participants could observe and exchange messages with what seemed to be other participants. In fact, the chat messages were pre-programmed and generated by bots, creating an illusion of a live, multi-person study environment. Based on post hoc participant feedback (see the Supplemental Materials), this deception appeared to be effective in sustaining the credibility of the experimental scenario.

**Figure 3.**
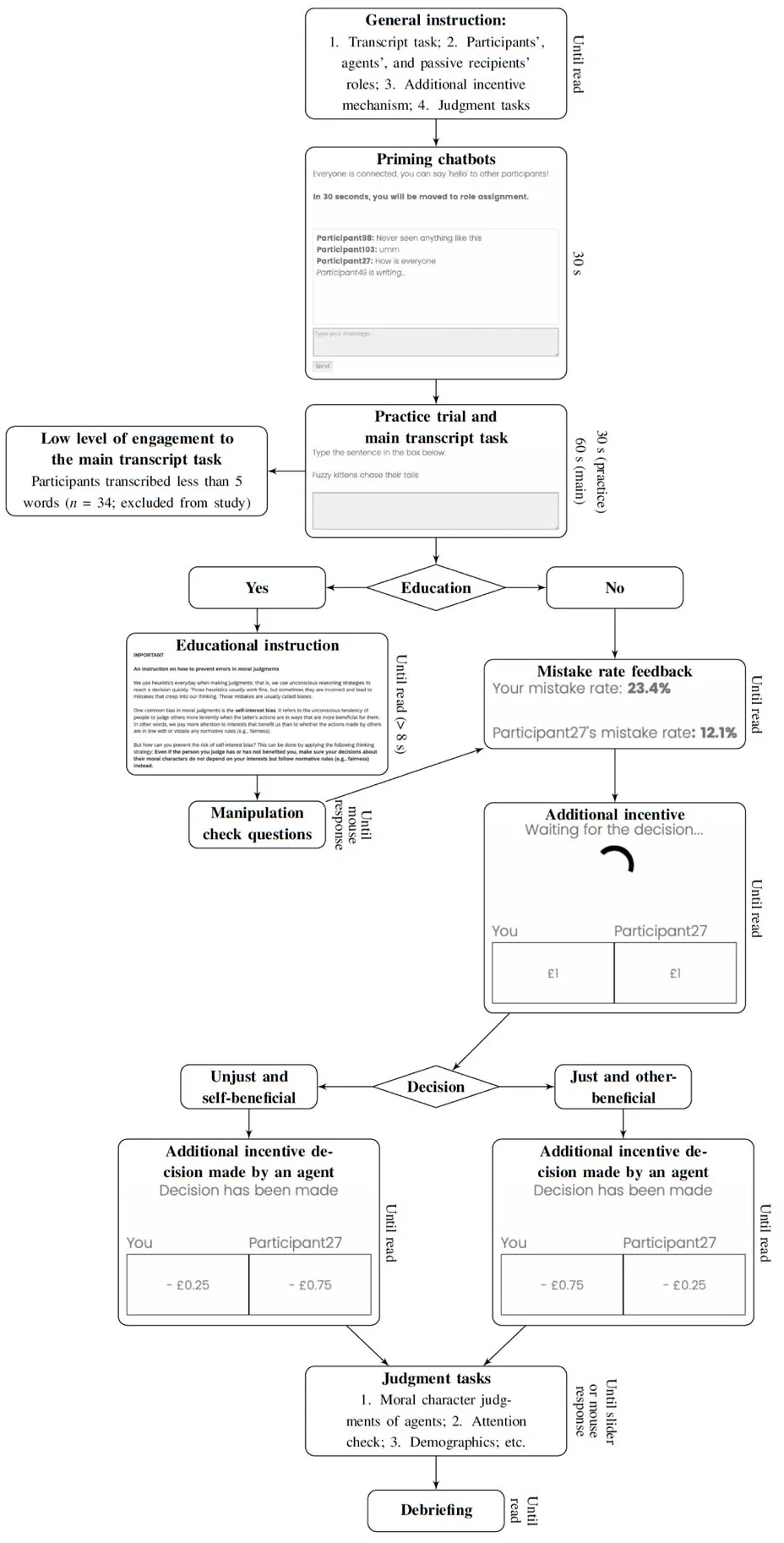
Flowchart of the procedure in Study 1. *Note*. The flow of information throughout the study is depicted using arrows, representative screenshots, and time durations. In the screenshots, “You” refers to the actual participant, while “Participant27” represents a fictitious participant controlled by the experimenter. For the full content of the educational instruction, please refer to the Method and Materials Supplement.

Next, all participants completed a 30-s practice trial followed by a 1-min main task. In both tasks, they were instructed to transcribe best-known tongue twisters (e.g., *She sells sea shells by the sea shore*) by typing as many words as possible in the correct sequence. Participants assigned to the education condition then read the instructional text on the self-interest bias and completed the corresponding manipulation check questions. Upon completion of the main task, an ex-ante unanimous mistake rate was appointed: all participants were told that they made errors at a rate of 23.4%, while the other participant (controlled by the experimenter) made nearly half fewer mistakes (12.1%). Based on this scenario, in the *unjust but self-beneficial* condition, participants received a smaller penalty (−£0.25), whereas the other participant received a larger penalty (−£0.75). By contrast, in the *just but other-beneficial* condition, participants received the larger penalty (−£0.75), and the other participant received the smaller one (−£0.25; see Figure 3 for a flowchart of the procedure). Following the decider’s allocation, participants were asked to evaluate the moral character of the decider. Finally, all participants were thoroughly debriefed regarding the nature and purpose of the study.

### Measures

**Moral character judgments** were measured with five items: “This decider is just,” “. . . is moral,” “. . . is reliable,” “. . . is honest,” and “This is a trustworthy decider.” Participants indicated the extent to which they agreed that the agent has these traits using a 7-point scale ranging from 1 = *I strongly disagree* to 7 = *I strongly agree*, with 4 representing a neutral rating in these dimensions. These items were adapted from the communion morality facet of the Agency-Communion-Inventory (Abele et al., 2016) and were averaged to create an integrative measure of moral character judgments (α = .96, *M* = 4.2, *SD* = 1.6). Participants were presented with those five items per block via item randomization.

In addition to the measures for the manipulation check and the moral character judgments, we also included a couple of other measures (e.g., liking, fairness) in this and the subsequent studies for exploratory purposes. To keep the “Design” and “Results” sections as concise as possible, we do not report these measures here, but all exploratory variables can be found in the datasets of the studies that we provide in the OSF repository (https://osf.io/gnz7c; cf., the Supplemental Materials and Method and Materials Supplement).

### Results

#### Manipulation Check

Binomial tests showed that for both educational statements included as a manipulation check, the percentage of participants who chose the answer “True” (93.1% and 96.0%) was significantly greater than the percentage of participants who chose the alternative answer “False”, *p*s < .001, *g*s = 0.46 and 0.48, respectively. These results provide evidence that the educational intervention was successful in conveying its intended message and that participants understood and accepted the core principles underlying the manipulation.

#### Moral Character Judgments

**Figure 4.**
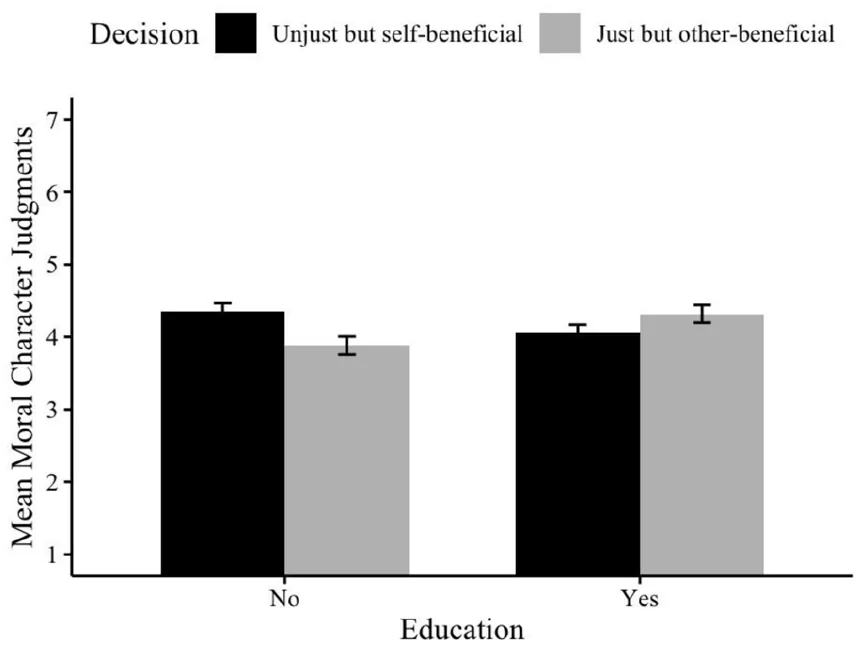
Moral character judgments as a function of education (Study 1). *Note*. Error bars are the ±1 standard error of the mean.

A 2 (Decision: Unjust but Self-Beneficial vs. Just but Other-Beneficial) × 2 (Education: Yes vs. No) analysis of variance (ANOVA) revealed a significant interaction effect, *F*(1, 684) = 9.41, *p* = .002, η*²*_p_ = 0.014, 95% CI [−0.90, 0.87], consistent with our hypothesis (see Figure 4). Pairwise comparisons showed that, in the non-education condition, participants whose self-interest was served by the unjust decision judged the decider more moral (*M* = 4.35, *SD* = 1.53) than did those whose self-interest was harmed in the just decision (*M* = 3.88, *SD* = 1.63), Welch’s *t*(338.7) = 2.73, *p* = .007, *d* = 0.30, 95% CI [0.13, 0.80]. By contrast, in the education condition, the effect of self-interest on moral character judgments was eliminated; participants whose self-interest was aided judged the decider similarly (*M* = 4.06, *SD* = 1.41) to those whose self-interest was harmed (*M* = 4.32, *SD* = 1.62), Welch’s *t*(338.7) = 1.58, *p* = 0.116, *d* = 0.17, 95% CI [−0.04, 0.38]. There were no main effects of self-interest, *F*(1, 684) = 0.76, *p* = .383, η*²*_p_ = 0.001, 95% CI [−0.34, 0.13], and education, *F*(1, 684) = 0.42, *p* = .518, η*²*_p_ = 0.001, 95% CI [−0.16, 0.31]. Besides, we reran our analysis while excluding those participants who incorrectly answered the two manipulation check questions in the education condition (*n* = 24). We found the same pattern of results as for analyzing the full dataset (see the Supplemental Materials).

## Discussion

As anticipated, Study 1 demonstrated that educating individuals about the self-interest bias in moral character judgments promoted more objective evaluations, rendering judgments less dependent on participants’ personal interests. By contrast, participants in the non-education condition—consistent with prior research—rated agents as more moral when their decisions aligned with the participants’ own interests, even if those decisions were unjust, and as less moral when the decisions were just but personally disadvantageous. These findings support Hypothesis 1 and suggest that increasing awareness of how self-interest can distort moral evaluations may serve as an effective debiasing strategy. The success of this educational intervention implies that it may work by shifting individuals’ motivations, enhancing recognition of fairness norms, and fostering a value orientation that prioritizes impartial justice over self-interest. Building on these findings, Study 2 examined whether encouraging greater cognitive effort could similarly attenuate the self-interest in moral character judgments.

## Study 2

Study 2 tested Hypothesis 2, which proposed that holding individuals accountable for the process of their moral character judgments by requiring written justifications would reduce or eliminate the self-interest bias. We expected that in the *unaccountable* condition, the self-interest bias would manifest: participants would judge an agent who made unfair decisions that benefited them as more moral than an agent who made fair decisions that advantaged another person. By contrast, in the *accountable* condition, we hypothesized that this bias would be attenuated or eliminated, such that participants would evaluate an agent making unjust self-serving decisions as no more moral than those making just, other-serving decisions. The hypothesis, method, statistical analysis, and sample size determination were preregistered at https://aspredicted.org/4vd_k6x.

### Method

#### Participants

To determine a priori sample size, we referred to the small interaction effect size observed in Study 1 (*f* = 0.12) and conducted an a priori power analysis using G*Power (Faul et al., 2007). The analysis indicated that a total sample of 917 participants would be needed to detect this effect with adequate power. To account for potential exclusions, we aimed to recruit at least 1,000 participants (approximately 250 per condition). Ultimately, 1,022 participants from the United States were recruited. Following the exclusion of participants who devoted a low level of engagement in the task (*n* = 54) and failed the attention check (*n* = 1), the final sample, therefore, included *N* = 967 participants (*M*_age_ = 37.7 years, *SD* = 10.9; 49.1% female; 49.3% male; 1.6% unspecified).

#### Design and Procedure

Participants were randomly assigned to one of four conditions according to a 2 (Decision: Unjust but Self-Beneficial vs. Just but Other-Beneficial) × 2 (Accountability: Accountable vs. Control) between-subjects design. With a few exceptions described below, the design and procedure were similar to those in Study 1.

Participants in the *accountable* condition were informed at the outset that, upon completion of the experimental tasks, we would request them to share their impressions of other participants to help us better understand how people form opinions about others. After the mistake rate and the decision (either unjust but self-beneficial or just but other-beneficial) were presented, participants were reminded that they would be required to justify the reasons behind their judgments (process accountability) at the end of the study. In doing so, they provided written justifications. By contrast, participants in the *control* condition were informed at the outset that their responses would be treated anonymously and confidential and would not be accessible to the researchers (see the Supplemental Materials for the full description of the accountability manipulation and justification analyses).

### Measures

Moral character judgments were measured as in Study 1 (α = .95, *M* = 4.2, *SD* = 1.5).

### Results

A 2 (Decision: Unjust but Self-Beneficial vs. Just but Other-Beneficial) × 2 (Accountability: Accountable vs. Control) ANOVA revealed a significant main effect of decision, *F*(1, 963) = 4.37, *p* = .037, η*²*_p_ = 0.005, 95% CI [−0.40, 0.01]. Participants rated the agent as more moral in the unjust but self-beneficial condition (*M* = 4.33, *SD* = 1.57) than in the just but other-beneficial condition (*M* = 4.13, *SD* = 1.52), Welch’s *t*(962.0) = 2.09, *p* = .037, *d* = 0.14, CI 95% [0.02, 0.40]. There was no main effect of accountability, *F*(1, 963) = 0.00, *p* = .999, η*²*_p_ = 0.000, 95% CI [−0.20, 0.20], nor a significant interaction effect, *F*(1, 963) = 1.60, *p* = .206, η*²*_p_ = 0.002, 95% CI [−0.68, 0.95]. Contrary to our prediction, Hypothesis 2 was not supported.

## Discussion

In Study 2, contrary to our expectations and to the general trend in which accountability reduces cognitive biases, we found that accountability did not eliminate the influence of self-interest on moral character judgments. Nevertheless, the impact of self-interest on such judgments was confirmed. These results suggest that individuals may still maintain biased judgments even under accountability pressure, potentially by drawing on justificatory or rationalization processes, as proposed by various motivated reasoning accounts (Darke & Chaiken, 2005; Ditto et al., 2009; Shalvi et al., 2012). However, several limitations may have reduced the effectiveness of our accountability manipulation. Notably, prior research on accountability has typically involved laboratory settings where participants interact with an experimenter in person (e.g., Simonson & Nye, 1992). This raises doubts about whether our online manipulation could successfully elicit comparable expectations of justification.

Furthermore, although our self-interest manipulation was behavioral, it was brief and somewhat ambiguous, potentially allowing participants to interpret unjust decisions as fair. This ambiguity may have enabled them to justify biased judgments. Supporting this interpretation, additional analyses showed that participants in the unjust but self-beneficial condition perceived the decision as fairer than those in the just but other-beneficial condition (see the Supplemental Materials). In addition, because people are generally averse to losses (Tversky & Kahneman, 1986), penalizing participants for mistakes may have evoked a sense of unfair economic harm, further facilitating justification for biased judgments. In Study 3, we sought to address these limitations by refining the self-interest manipulation.

Finally, we suspect that the absence of an accountability effect in reducing the self-interest bias may be partially attributed to the way that confidentiality was emphasized in the unaccountable condition. Specifically, participants were explicitly reminded, beyond the standard informed consent, that their responses would remain anonymous and confidential. By contrast, unaccountable control conditions in laboratory settings often imply anonymity without overemphasizing it, typically using face-to-face interviews where such assurances are less formalized (e.g., Schulz-Hardt et al., 2021). Research has shown that excessive assurances of anonymity and confidentiality can have an opposite effect, potentially undermining the perceived credibility of the experimenter and reducing participants’ willingness to respond truthfully (Rasinski et al., 1999). To address this concern, we modified the procedure in Study 3 by restricting all anonymity and confidentiality information to the initial informed consent stage only. We further discuss this issue—namely, respondent anonymity in online studies—as a potential moderator of accountability effects in the General Discussion.

## Study 3

In Study 3, we re-examined our hypothesis that holding individuals accountable for their moral judgments would either reduce or completely eliminate the self-interest bias. To this end, we employed a refined self-interest manipulation, differing from Study 2. Specifically, participants read hypothetical moral vignettes in which a target person transgressed across various social contexts (e.g., examination, politics) and either served or did not serve the participants’ personal interests. Additionally, we strengthened the accountability manipulation by informing participants that they would be interviewed by the experimenter at the end of the study to justify their judgments in an audio-recorded session. To assess the effectiveness of this manipulation, we also measured the extent to which participants believed that they would actually meet with the experimenter. The hypothesis, method, statistical analysis, and sample size determination were preregistered at https://aspredicted.org/1gq_thb.

### Method

#### Participants

To determine a priori sample size, we referred to a moderate main effect of the self-interest manipulation (*f* = 0.27) observed in an unpublished study that employed the same set of moral vignettes as used in the present study. A power analysis conducted in G*Power indicated that a minimum sample of 152 would be required to detect this effect with adequate power. Following the recommendations by Giner-Sorolla et al. (2018) to ensure robust estimation, we multiplied this base by four, resulting in a planned sample size of 608. Ultimately, we recruited 648 participants from the United States. Data from four participants who failed two comprehension check questions (one per vignette) were excluded to ensure sufficient comprehension of the scenarios. The final sample thus comprised *N* = 644 participants (*M*_age_ = 39.8 years, *SD* = 29.5; 50.0% female; 48.9% male; 1.1% unspecified).

#### Design and Procedure

Participants were randomly assigned to one of four experimental conditions in a 2 (Self-Interest: Involved vs. Noninvolved) × 2 (Accountability: Accountable vs. Control) between-subjects design. Within each condition, participants were further randomly assigned to read two of four vignettes, each depicting a different moral agent (a student, a member of parliament, a line manager, or a software user) who engaged in transgressive behavior across various social domains (cheating on an exam, accepting bribes, or violating software copyright; see the Supplemental Materials for full vignette texts). Each vignette appeared in one of two versions: one in which the participants’ self-interest was presumably involved, and one in which it was not. To ensure data quality, participants were asked to complete a comprehension check immediately after reading each vignette. Participants who failed both comprehension checks were excluded from further analysis.

Participants in the *accountable* condition were informed that, upon completing the experimental tasks, they would take part in an online audio-recorded interview in which an experimenter would ask them to explain the reasons behind their moral character judgments (i.e., process accountability). This information was conveyed during the informed consent process and reiterated in the instructional materials prior to participants making any judgments. This manipulation resembled the approach utilized in Study 2, with the key distinction being that accountability was framed as an audio-based rather than written process. Prior studies suggest that oral argumentation enhances reasoning, including the generation of arguments, rebuttals, and the integration of textual information (Reznitskaya et al., 2001). Therefore, we anticipated that this refinement would strengthen the effectiveness of the accountability manipulation.

By contrast, participants in the *control* condition received no mention of an interview or evaluation of their judgments. All participants then rated the moral character of the transgressor using the same measures as in Study 2. To assess the effectiveness of the accountability manipulation, participants were asked, after completing all measures but prior to debriefing, how strongly they anticipated being interviewed by the experimenter.

### Measures

**Moral character judgments** were measured as in Studies 1 and 2 (α = .93, *M* = 2.9, *SD* = 1.2).

**Accountability expectation** was measured with one item: “Do you expect to explain the reasons for your judgments to an experimenter?”, on a 7-point scale where 1 = *Definitely not* and 7 = *Definitely yes*, with the middle point 4 = *I am not sure*.

### Results

#### Accountability Expectation

A 2 (Self-Interest: Involved vs. Noninvolved) × 2 (Accountability: Accountable vs. Control) ANOVA revealed a significant main effect of accountability, *F*(1, 640) = 56.36, *p* < .001, η*²*_p_ = 0.081, CI 95% [0.66, 1.13]. Participants in the accountable condition expected more to explain the reasons for their judgments to the experimenter (*M* = 5.50, *SD* = 1.41) than those in the control condition (*M* = 4.61, *SD* = 1.53), Welch’s *t*(574.3) = 7.62, *p* < .001, *d* = 0.44, CI 95% [0.66, 1.13]. The results provided evidence that our experimental instructions and manipulations primed accountability effectively.

#### Moral Character Judgments

For each participant, we averaged the scores of their moral character judgments of the transgressor across the two vignettes and submitted them to a 2 (Self-Interest: Involved vs. Noninvolved) × 2 (Accountable vs. Control) ANOVA. This analysis yielded a significant main effect of self-interest, *F*(1, 640) = 24.29, *p* < .001, η*²*_p_ = 0.037, CI 95% [0.27, 0.63]. Participants whose self-interest was involved judged the transgressor more moral (*M* = 3.10, *SD* = 1.16) than did those whose self-interest was noninvolved (*M* = 2.73, *SD* = 1.08), Welch’s *t*(636.2) = 4.93, *p* < .001, *d* = 0.39, CI 95% [0.27, 0.63]. There was no main effect for the accountability, *F*(1, 640) = 0.21, *p* = .644, η*²*_p_ = 0.000, CI 95% [−0.23, 0.14], nor was there an interaction effect, *F*(1, 640) = 0.11, *p* = .744, η*²*_p_ = 0.000, CI 95% [−0.42, 0.84]. A Pearson correlation test revealed that moral character judgments were completely uncorrelated with the accountability expectation, *r* = −.04, *t*(642) = −1.10, *p* = .273.

## Discussion

Study 3 replicated the findings of Study 2 using a different self-interest manipulation and a more rigorous process accountability intervention. As in Studies 1 and 2, participants’ moral character judgments were influenced by whether their self-interest was implicated. Contrary to our hypothesis—yet consistent with the results of Study 2—process accountability did not attenuate the self-interest bias. Notably, the accountability manipulation check confirmed the credibility and effectiveness of the intervention, raising important questions about the efficacy of process accountability as a debiasing strategy. These findings cast doubt on whether process accountability alone is sufficient to reduce the self-interest bias. In Study 4, we sought to target motivated reasoning more directly by instructing participants to base their judgments on social and ethical norms, for which they would be held accountable.

## Study 4

The previous two studies yielded judgment patterns that ran counter to our predictions. To further assess the diagnostic strength of the accountability manipulation, we conducted a replication Study 4 that focused exclusively on accountable conditions. Therefore, Study 4 replicated Study 3 but implemented more rigorously constructed instructions tailored to each condition. Drawing on the successful education manipulation from Study 1, in which participants were taught to base their moral character judgments on normative standards such as fairness rather than personal interests, we modified the process accountability manipulation to explicitly emphasize justifications according to social and ethical norms. This enhancement was intended to bolster the effectiveness of the process accountability intervention by fostering participants to focus on the normative reasoning process underlying their judgments. All other procedural elements in Study 4 remained consistent with those in Study 3. The hypothesis, method, statistical analysis, and sample size determination were preregistered at https://aspredicted.org/rq8_z7v.

### Method

#### Participants

We used the same approach as in Study 3 for the sample estimation. We recruited 703 participants from the United States. Data from 75 of these participants who failed one vignette comprehension check question were excluded from further analysis. Our final sample, therefore, comprised *N* = 628 participants (*M*_age_ = 39.8 years, *SD* = 13.0; 49.8% female; 48.7% male; 1.5% unspecified).

#### Design and Procedure

The design and procedure were largely identical to those used in Study 3, with two key exceptions. First, instead of the control accountability condition, we introduced a new accountability condition—accountable to norms of justice—in which participants were instructed to “justify the reasons for their judgments according to social and ethical standards such as norms of justice” (see the Method and Materials Supplement for exact wording). Therefore, the study employed a 2 (Self-Interest: Involved vs. Uninvolved) × 2 (Accountability: Accountable to Norms of Justice vs. Only Accountable). Participants in the *accountable to norms of justice* condition were explicitly directed to ground their justifications in social and ethical norms (e.g., justice), whereas those in the *only accountable* condition were asked to justify their judgments without reference to any normative standards. Second, participants were randomly assigned to read only one of the four vignettes, rather than two as in Study 3.

### Measures

**Moral character judgments** were measured as in Studies 1 through 3 (α = .95, *M* = 3.2, *SD* = 1.6).

**Accountability expectation** in the *accountable to norms of justice* condition was measured with one item: “Do you expect to explain the reasons for your judgments to an experimenter according to social and ethical norms such as norms of justice?” In the *only accountable* condition, the item was: “Do you expect to explain the reasons for your judgments to an experimenter?”, as used in Study 3. Both items were presented in a bipolar format on a 7-point scale (1 = *Definitely not*; 4 = *I am not sure*; 7 = *Definitely yes*), consistent with the scale used in Study 3.

### Results

#### Accountability Expectation

The binomial tests show that, in the *accountable to norms of justice* condition, the percentage that participants expected to explain the reasons for their judgments according to social and ethical norms such as norms of justice (76.9%; i.e., those ratings to the corresponding accountability expectation question > 4) was significantly larger than the percentage that participants did not expect so (7.9%; i.e., those ratings < 4), *p* < .001, *g* = 0.27. In a similar vein, in the *only accountable* condition, the percentage that participants expected to explain the reasons for their judgments (83.7%) was significantly larger than the percentage that participants did not expect so (7.0%), *p* < .001, *g* = 0.34. The results again proved that our experimental instructions and manipulations primed accountability effectively. There was no difference between these two groups in participants’ expectations to be held accountable, *p* = .102.

#### Moral Character Judgments

Confirming the findings of Study 3, we found a main effect of self-interest, *F*(1, 624) = 9.74, *p* = .002, η*²*_p_ = 0.015, 95% CI [0.14, 0.63]. Participants whose self-interest was involved judged the agents more moral (*M* = 3.36, *SD* = 1.53) than did those whose self-interest was noninvolved (*M* = 2.97, *SD* = 1.56), Welch’s *t*(619.6) = 3.12, *p* = .002, *d* = 0.25, CI 95% [0.14, 0.63]. There was no main effect of accountability, F(1, 624) = 0.01, p = .929, η*²*_p_ = 0.000, 95% CI [−0.23, 0.25], nor was there an interaction effect, F(1, 624) = 0.32, p = .574, η*²*_p_ = 0.001, 95% CI [−0.49, 0.90]. Pearson correlation tests revealed that moral character judgments were completely uncorrelated with the *only accountable* expectation, *r* = .01, *t*(311) = 0.15, *p* = .878, but were weakly correlated with the *accountable to norms of justice* expectation, *r* = −.12, *t*(313) = −2.18, *p* = .030. This correlation was supported by exploratory analyses, showing that, in the *accountable to norms of justice* condition, moral character judgments of those whose self-interest was involved (*M* = 3.33, *SD* = 1.45) versus those uninvolved (*M* = 3.02, *SD* = 1.49) just slightly missed the significance level of difference, *F* = 3.63, *p* = .058, η*²*_p_ = 0.01.

## Discussion

We examined whether enhancing process accountability through an educational intervention—specifically, by instructing participants to justify their judgments according to social and ethical norms, such as norms of justice (vs. merely being held accountable) —would eliminate the impact of self-interest on moral character judgments. Consistent with findings from Studies 2 and 3, process accountability, even when normatively framed, did not reduce the self-interest bias. Thus, across multiple studies employing varied manipulations of both self-interest and accountability, we found no evidence that accountability alone effectively debiases moral character judgments shaped by self-interest. In our final study, we therefore tested whether a joint intervention that combined education with accountability might more successfully mitigate the self-interest bias.

## Study 5

Building on the unexpected patterns observed in the previous three studies, Study 5 sought to replicate and extend those earlier findings by simultaneously testing the individual and combined effects of two debiasing strategies: education and accountability. Specifically, we investigated whether educating participants about the self-interest bias and/or holding them accountable for their moral character judgments would reduce the extent to which self-interest influences those judgments. We predicted that participants in the education (vs. non-education) condition and/or in the process or outcome accountability (vs. control unaccountable) conditions would show a reduced self-interest bias. In particular, when an agent’s unethical action served participants’ self-interest, they would judge the agent’s morality similarly to (vs. more positively than) those whose self-interest was not served by the same action. All hypotheses, methods, statistical analyses, and sample size determination were preregistered at https://aspredicted.org/r7kv-rg89.pdf.

### Method

#### Participants

To determine a priori sample size, we referred to the effect sizes observed in Study 3 (η*²*_p_ = 0.037) and in Study 4 (η*²*_p_ = 0.015) for the main effect of self-interest. Averaging these estimates yielded η*²*_p_ = 0.026, corresponding to an effect size of *f* = 0.16. A power analysis conducted using G*Power indicated that, to detect this effect with 95% power in a between-subjects ANOVA, a total sample size of 582 would be required. To account for potential exclusions due to failed attention checks, as observed in Studies 3 and 4, we recruited 648 participants from the United States. Data from seven of these participants who failed two check questions (one question per vignette) were excluded from further analysis. Our final sample, therefore, consisted of *N* = 641 participants (*M*_age_ = 41.3 years, *SD* = 13.0; 49.9% female; 49.9% male; 0.2% unspecified).

#### Design and Procedure

Participants were randomly assigned to one of twelve conditions in a 2 (Self-Interest: Involved vs. Not Involved) × 2 (Education: Yes vs. No) × 3 (Accountability: Process vs. Outcome vs. Control) between-subjects design. The *education* manipulation replicated that of Study 1. In all *accountability* conditions, participants were informed that they would undergo an online audio interview by an experimenter upon completing the experimental tasks. Specifically, in the *process* accountability condition, they were told that they would need to justify how they reached their moral judgments, detailing their reasoning process and decision steps. In the *outcome* accountability condition, they were informed that they would need to justify the accuracy or effectiveness of their moral judgments, the quality of which would be evaluated relative to others’ responses. The *control* condition and general procedural elements were identical to those used in Study 3.

### Measures

**Moral character judgments** were measured as in Studies 1 through 4 (α = .96, *M* = 2.9, *SD* = 1.3).

**Education manipulation check** was measured as in Study 1.

**Accountability expectation** was measured as in Study 3.

### Results

#### Education Manipulation Check

The binomial tests showed that for the two educational statements regarding the manipulation check of the education instruction (cf., the “Design” section in Study 1), the percentages that the answer “True” was chosen (95.4% and 98.5%) were significantly greater than the percentages that the alternative answer “False” was chosen, *p*s < .001, *g*s = 0.45 and 0.48, respectively, providing evidence that this was a successful manipulation of education on the self-interest bias in moral character judgments.

#### Accountability Expectation

A 2 (Self-Interest: Involved vs. Not Involved) × 3 (Accountability: Process vs. Outcome vs. Control) ANOVA revealed a significant main effect of accountability, *F*(1, 633) = 48.45, *p* < .001, η*²*_p_ = 0.132, CI 95% [−0.22, 1.55]. Participants in both the process and outcome accountability conditions expected more to explain the reasons for their judgments to the experimenter (*M*_process_ = 5.90, *SD* = 1.31; *M*_outcome_ = 5.76, *SD* = 1.49) than did those in the control condition (*M* = 4.68, *SD* = 1.54), Welch’s *t*(412.5)_process_ = 9.02, *p* < .001, *d* = 0.84, CI 95% [0.95, 1.49]; Welch’s *t*(403.1)_outcome_ = 7.54, *p* < .001, *d* = 0.71, CI 95% [0.80, 1.36]. There was no difference between the process and outcome accountability conditions in participants’ expectations to be held accountable, Welch’s *t*(356.2) = 0.95, *p* = .344, *d* = 0.10, CI 95% [−0.15, 0.43]. These results provided evidence that our experimental instructions and manipulations effectively primed accountability.

#### Moral Character Judgments

Consistent with the findings of Studies 3 and 4, we found a main effect of self-interest, *F*(1, 628) = 13.53, *p* < .001, η*²*_p_ = 0.021, 95% CI [0.17, 0.56]. Participants whose self-interest was involved judged the agents more moral (*M* = 3.04, *SD* = 1.34) than did those whose self-interest was noninvolved (*M* = 2.67, *SD* = 1.20), Welch’s *t*(630.8) = 3.63, *p* < .001, *d* = 0.29, CI 95% [0.17, 0.56]. There was no main effect of education, F(1, 628) = 1.69, p = .195, η*²*_p_ = 0.002, 95% CI [−0.07, 0.33], nor accountability, F(1, 627) = 2.62, p = .074, η*²*_p_ = 0.009, 95% CI [−0.16, 0.57], nor was there an interaction effect of self-interest, education, and accountability, F(1, 627) = 0.12, p = .891, η*²*_p_ = 0.000, 95% CI [−0.71, 1.06]. A Pearson correlation test revealed that moral character judgments were completely uncorrelated with the accountability expectation, *r* = −.01, *t*(639) = −0.29, *p* = .771.

## Discussion

The findings from this study suggest that neither the educational intervention nor the accountability manipulations, whether process- or outcome-based, produced significant main effects, nor did they interact with self-interest. These null effects, along with Studies 2 through 5, raise important questions about the effectiveness of these commonly studied debiasing strategies. Notably, the absence of a relationship between participants’ accountability expectations and their moral character judgments suggests that simply informing them of upcoming justification demands may not be sufficient to alter their evaluative processes, especially when the bias operates implicitly. While prior work and Study 1 have highlighted the potential of education to mitigate biased reasoning, these results suggest that awareness alone may not be enough to override self-serving tendencies in moral contexts.

## General Discussion

In five preregistered studies, we examined two theoretically distinct interventions aimed at reducing the self-interest bias in moral character judgments: education, designed to shift moral motivation and encourage impartiality (Studies 1, 4, and 5), and accountability—either process or outcome—intended to enhance cognitive effort and scrutiny (Studies 2 through 5). While our accountability manipulations were systematically refined to heighten participants’ sense of being held accountable, we found no consistent evidence that accountability attenuated the self-interest bias. By contrast, Study 1 showed that the educational intervention successfully reduced the bias. However, this effect was not replicated in Study 5, revealing a lack of convergence in the literature and pointing to a broader challenge in reconciling findings on educational approaches to mitigating self-serving moral judgments. We suggest that these inconsistent results may be due to differences in experimental targets, design features, timing of interventions, measurement strategies, or stimuli used across studies. Below, we propose, to our knowledge, the most plausible explanation for the divergence in findings between Studies 1 and 5.

In Study 1, the self-interest manipulation involved an unjust but self-beneficial scenario, contrasted with a just but other-beneficial control condition. While these two conditions (i.e., self-beneficial vs. other-beneficial) are, inter alia, conceptually similar to the self-interest involved versus not involved manipulation used in Study 5, a key distinction lies in the moral valence of the transgressions. Specifically, all vignettes in Study 5 uniformly described unjust actions, whereas Study 1 included a just control condition. Recall that we operationalized the self-interest bias as the difference in participants’ moral character judgments of agents whose actions were unjust but beneficial to them versus agents whose actions—either just in Study 1 or unjust in Study 5—did not benefit them. This distinction suggests that the presence of a *just* action in the control condition of Study 1 versus the uniformly unjust actions in Study 5 may have had a more fundamental influence on participants’ moral judgments than the presence or absence of the educational intervention itself. The nuanced interplay between moral justness and self-interest, therefore, may be central to understanding the divergence in effects observed across studies.

Nevertheless, Study 1 suggests that, according to the focus theory of normative conduct (Cialdini et al., 1990), directing people’s attention primarily to descriptive norms—what the majority typically does—can significantly impact their moral evaluations of others and thus help mitigate cognitive biases. Then, introducing knowledge aimed at debiasing judgments enables individuals to incorporate new normative standards into their moral reasoning frameworks (Jiang et al., 2023). This aligns with prior research showing that educational interventions targeting cognitive biases can enhance individuals’ ability to recognize and mitigate those biases (Morewedge et al., 2015). Although it is plausible that individuals can be motivated to shift from prioritizing self-interest to guarding justice, the findings from Study 5 suggest that mere awareness of cognitive biases does not always lead to normatively appropriate decision-making (Pronin et al., 2004).

According to the social intuitionist model, moral judgments are primarily driven by intuitive, affective responses, with reasoning processes serving a post-hoc justificatory role (Haidt, 2001). That is, individuals often generate rational justifications after experiencing quick, gut-level reactions to moral situations, primarily to defend or legitimize those justifications. In this light, accountability may not counteract the self-interest bias; instead, it may trigger post-hoc rationalization, enabling individuals to devise self-serving explanations that seem reasonable yet ultimately preserve biased moral evaluations. This interpretation is consistent with research showing that justification processes can enable rather than reducing unethical behavior (Shalvi et al., 2012).

The social intuitionist model also argues that the justification that people offer for a moral judgment is often distinct from the actual source of that judgment (Haidt, 2001). Given that individuals are usually unaware of the self-interest bias influencing their evaluations (Bocian & Wojciszke, 2014b), this may explain why the accountability intervention consistently failed to attenuate the bias, whereas the educational intervention showed some success, albeit only partially. When unaware of the source of the bias, individuals can easily generate plausible post-hoc justifications that affirm their judgments as moral and fair. By contrast, when the bias is explicitly revealed such as through educational prompts, these justifications become harder to sustain, thereby increasing the likelihood of correction in line with justice norms. This reasoning aligns with classic findings on moral hypocrisy, which is reduced when individuals are prompted to compare their behavior against normative standards through self-awareness (Batson et al., 1999).

Interpreting the null effects of accountability requires critically assessing its theoretical and practical value as a debiasing tool. First, a convincing theory for the effect of accountability on reducing biases remains elusive. For example, one meta-analysis found that the effect of rater accountability on performance ratings varies greatly and depends on different characteristics of accountability source (e.g., whether it is a ratee or a superior who holds raters accountable), of manipulation type (e.g., identification or justification), of study setting (e.g., field or laboratory), and of rating direction (Harari & Rudolph, 2017). Another meta-analysis demonstrated that, as moderated by task complexity, accountability by itself cannot act as a powerful motivator of better performance (Sharon et al., 2022). Therefore, it is not unexpected that the current research found that process as well as outcome accountability fails to impact respondents’ moral attitudes positively and significantly.

Second, past research found that when agents are held accountable for their behaviors, their justice breaches are less frequently categorized in moral terms when moral reasoning, such as social domain categorizations, is tapped simultaneously with accountability appraisals rather than alone (Bersoff & Miller, 1993). This implies a sensitive gauge of how these two moral judgment processes could capture people’s attention to the scenarios. Then, based on this methodological effect on moral judgments, it is reasonable to formulate predictions that when only the accountability approach is assessed, people may be prone to withhold others’ moral blame, at least in some instances, without holding themselves accountable for those misbehaviors. In this sense, accountability alone is not a sufficient corrective mechanism; without an accompanying shift in motivation, it may serve as a tool for justification rather than for genuine bias reduction. Indeed, it has been noted that since task complexity moderates the interplay between accountability and judgmental or decision performance, accountability per se cannot serve as a sole motivator of better performance advantage (Sharon et al., 2022).

Third, we may assume that the self-interest bias is a form of defensive bias, meaning that individuals tend to align their attitudes with their self-interest rather than with objective reasoning. More importantly, research shows that when decisions or issues are personally relevant, it leads people to engage in more extensive reasoning due to increased cognitive processing, but it still remains self-serving reasoning (Darke & Chaiken, 2005). This may explain why our accountability interventions failed—that is, an increased cognitive effort does not mean that people stop focusing on personal costs and benefits.

Fourth, empirical findings suggest that *process* accountability exerts a beneficial performance effect in simple elemental tasks, with an emphasis on cue-criterion abstraction processing that is mentally integrated to form a judgment. By contrast, *outcome* accountability is exploited in more complex configurable tasks, with the use of exemplar-based processing that relies on episodic memory traces of exemplars (De Langhe et al., 2011). Recall that drawn on dual-process and fuzzy-trace theories, we argue that since individuals whose self-interest is involved are inclined to encode their judgments using Type 1 and gist processing, it is unlikely for accountable individuals to form analytical cue abstraction processing based on classical rationality that relates a trade-off of their self-interest to the agent’s behavior to be judged. Although our outcome accountability intervention in Study 5 appeared ineffective, it remains theoretically plausible that outcome-accountable individuals—depending on individual differences in analytical intelligence and memory retrieval—might evoke Type 2 processing and retrieve verbatim representations of their self-interest. This could occur through similarity-based reasoning, whereby individuals compare the wrongdoer’s behavior to concrete exemplars stored in episodic memory.

Fifth, unlike many earlier studies, participants on the Prolific platform may genuinely feel anonymous while making judgments. Previous research indicates that assurances of anonymity can lead to higher rates of nonresponse and misreporting in non-face-to-face surveys (Fuller, 1974). Consequently, anonymity may undermine accountability, leading participants to believe they do not need to respond to certain questions or that the accuracy of their answers is unimportant. Our participants may lack the epistemic motivation to respond to or even disregard our accountability concerns—regardless of the justification formats—when they are confident that our requirement to justify their judgments is not enforced, as long as the compensation structure remains unchanged. This conjecture may hint at selection favoring cuing effects such as accountability that uniformly ensures deliberative thinking in some environments. Therefore, predictions regarding the methodological effects of accountability on moral judgments should be formulated based on the relative adequacy of the procedures to capture people’s morally accountable pressure being held in specific contexts.

Finally, we manipulated participants in the United States in acute presumable conditions designed to instruct them on particular moral biases and how to avoid them and to hold them accountable for their judgments. However, the effectiveness of accountability interventions may be culture-dependent. In individualistic cultures, where self-justification is highly valued (Markus & Kitayama, 1991), justification-based accountability may encourage individuals to defend their biases rather than to correct them. By contrast, in collectivist cultures, where individuals are more attuned to group-based moral expectations (Miller & Bersoff, 1992), accountability mechanisms that emphasize collective responsibility and social reputation may be more effective. Research on whether accountability underlying collectivist (vs. individualistic) moral responsibility could enhance its effectiveness in bias reduction is still in scant.

### Implications

Our findings suggest that bias-reduction interventions must do more than simply demand justification—they must also shift moral motivation. In professional, legal, and policy settings, accountability mechanisms should be structured to avoid reinforcing self-serving rationalizations. Rather than relying on self-justification alone, accountability mechanisms should incorporate peer evaluation, hierarchical oversight, and public scrutiny to increase the cost of biased moral reasoning. These interventions may help ensure that accountability does not merely facilitate rationalization but instead promotes genuine moral reflection and fair decision-making.

The findings also speak to broader societal issues. That accountability as a corrective mechanism failed to moderate the self-interest bias raises questions about whether public scrutiny alone is an effective deterrent against unethical decision-making. If individuals can rationalize their biases even when they are required to justify their actions, then transparency measures, oversight mechanisms, and ethical reviews must be designed with an awareness of how self-serving justifications operate. Education may be a more effective means of fostering ethical decision-making in professional and public life. This suggests that policies aimed at reducing the self-interest bias should focus not just on holding individuals accountable after the fact but instead on proactively shaping moral motivation before decisions are made.

### Limitations

The results of this research are subject to several limitations. First, the recency of the educational intervention, which was delivered before participants made moral judgments, may have enhanced its effectiveness in Study 1. Second, in Studies 1 and 2, the somewhat unmatched ratios of pseudo-errors and penalties between participants and their counterparts in the main task may have alerted participants to inconsistencies between performance and outcomes, potentially influencing their perceptions of fairness. Although we attempted to account for potentially biased data patterns caused by the methodology employing the ex-ante unanimous mistake rate as a control variable (for details, see the Supplemental Materials), residual bias cannot be fully ruled out. Third, although the transcript task used in Studies 1 and 2 was novel, its framing of the decider and other participants as online peers may have shaped participants’ responses in ways not captured by our analyses. As such, potential confounding variables may still remain. Fourth, although the use of hypothetical moral vignettes is well-established in moral psychology research, this method is not without limitations and criticism.

### Future Directions

Future research should explore whether more robust forms of accountability, such as justification to an external audience (e.g., public reporting, hierarchical oversight, or peer evaluation) or scenarios with real-world reputational consequences, can encourage individuals to genuinely reevaluate their moral judgments rather than merely to defend them. Testing accountability in higher-stakes environments, where biased reasoning may carry tangible costs, could help determine the conditions under which accountability functions as a true debiasing mechanism rather than a justification tool. Beyond education and accountability, future research should explore alternative mechanisms for bias reduction. One promising approach is social norm interventions, where individuals observe unbiased decision-making in peers. Research on norm transmission suggests that people adjust their moral evaluations based on perceived social expectations and peer behavior (Cialdini et al., 1990). When individuals witness others engaging in impartial moral reasoning, they may internalize fairness norms and become less prone to self-serving biases (Tankard & Paluck, 2016). This aligns with work demonstrating that descriptive norms—perceptions of what others do—can significantly shape ethical decision-making (Hertwig & Grüne-Yanoff, 2017).

## Conclusion

In conclusion, we argue that self-interest, *Homo economicus*, should be regarded as a pervasive influence in moral character judgments. Across five preregistered studies, we examined the potential for educational and accountability-based interventions to mitigate the self-interest bias. Our findings suggest that while educational interventions may partially succeed—likely by increasing awareness and encouraging norm-based reasoning—accountability alone did not reliably reduce the self-interest bias. These results indicate that invoking cognitive effort through accountability may not be sufficient to override deeply rooted motivational influences. Instead, they highlight the robustness of the self-interest bias and emphasize the need for motivational strategies in promoting impartial moral judgments. Taken together, these findings underline that the cognitive and motivational mechanisms underlying the self-interest bias remain insufficiently understood. They also underscore the challenge of reducing this bias in moral character judgments and call for future work to explore alternative interventions that more directly target the motivational and affective mechanisms.

## Supplemental Material

sj-docx-1-psp-10.1177_01461672251364308 – Supplemental material for Testing Education and Accountability as Moderators of the Self-Interest Bias in Moral Character Judgments Supplemental material, sj-docx-1-psp-10.1177_01461672251364308 for Testing Education and Accountability as Moderators of the Self-Interest Bias in Moral Character Judgments by Yong Lu, Katarzyna Miazek and Konrad Bocian in Personality and Social Psychology Bulletin

sj-docx-2-psp-10.1177_01461672251364308 – Supplemental material for Testing Education and Accountability as Moderators of the Self-Interest Bias in Moral Character Judgments Supplemental material, sj-docx-2-psp-10.1177_01461672251364308 for Testing Education and Accountability as Moderators of the Self-Interest Bias in Moral Character Judgments by Yong Lu, Katarzyna Miazek and Konrad Bocian in Personality and Social Psychology Bulletin
