# Supplementary material for: Testing Education and Accountability as Moderators of the Self-Interest Bias in Moral Character Judgments
Source: Pers Soc Psychol Bull. 2025 Aug 28;52(10):3127–43. doi: 10.1177/01461672251364308 (PMC13507493; doi:10.1177/01461672251364308)
Supplement: Supplementary file 1 [file sj-docx-1-psp-10.1177_01461672251364308.docx]

# Method/Materials File for:

“Testing Education and Accountability as Moderators of the Self-Interest Bias in Moral Character Judgments”

*Note*. Raw datasets and R Notebooks (statistical analyses results) for each study are available on

| the Open Science Framework at <https://osf.io/gnz7c>. |  |
| --- | --- |
| **Contents** |  |
| **Contents** | **i** |
| **List of Tables** | **vi** |
| **List of Figures** | **vii** |
| **1 Method** | **1** |
| 1.1 Overview of the present studies . . . . . . . . . . . . . . . . . . . . . . . . . . . . | 1 |
| 1.2 Exploratory variables . . . . . . . . . . . . . . . . . . . . . . . . . . . . . . . . . | 1 |
| **2 Materials** | **6** |
| 2.1 Study 1 . . . . . . . . . . . . . . . . . . . . . . . . . . . . . . . . . . . . . . . . | 6 |
| 2.1.1 General introduction (all conditions) . . . . . . . . . . . . . . . . . . . . . | 6 |
| 2.1.2 General introduction (cont.; all conditions) . . . . . . . . . . . . . . . . . | 6 |
| 2.1.3 Message to participants who devoted a low level of engagement to the |  |
| main transcript task . . . . . . . . . . . . . . . . . . . . . . . . . . . . . . | 9 |
| 2.1.4 Introduction (the *education* condition) . . . . . . . . . . . . . . . . . . . . | 9 |
| 2.1.5 Introduction (the *non-education* condition) . . . . . . . . . . . . . . . . . | 11 |
| 2.1.6 Introduction (cont.; the *unjust and self-beneficial* condition) . . . . . . . . | 11 |

|  | 2.1.7 | Introduction (cont.; the *just and other-beneficial* condition) . . . . . . . . . | 12 |
| --- | --- | --- | --- |
|  | 2.1.8 | Manipulation check questions (the *education* condition) . . . . . . . . . . | 12 |
|  | 2.1.9 | Measure of *liking toward agents* (all conditions) . . . . . . . . . . . . . . | 13 |
|  | 2.1.10 | Measure of *mind of agents* (all conditions) . . . . . . . . . . . . . . . . . . | 13 |
|  | 2.1.11 | Measure of *moral character judgments of agents* (all conditions) . . . . . . | 13 |
|  | 2.1.12 | Attention check question (all conditions) . . . . . . . . . . . . . . . . . . | 13 |
|  | 2.1.13 | Prompt (all conditions) . . . . . . . . . . . . . . . . . . . . . . . . . . . . | 13 |
|  | 2.1.14 | Measure of *satisfaction of agents’ decisions* (all conditions) . . . . . . . . | 13 |
|  | 2.1.15 | Measure of *fairness of agents’ decisions* (all conditions) . . . . . . . . . . | 14 |
|  | 2.1.16 | Measure of *intention of agents’ decisions* (all conditions) . . . . . . . . . . | 14 |
|  | 2.1.17 | Measure of *mental effort of participants* (all conditions) . . . . . . . . . . | 14 |
|  | 2.1.18 | Measure of *recalls of the mistake rates* (all conditions) . . . . . . . . . . . | 20 |
|  | 2.1.19 | Measure of *purpose of the study* (all conditions) . . . . . . . . . . . . . . . | 20 |
|  | 2.1.20 | Measure of *suspicions of the mistake rates* (all conditions) . . . . . . . . . | 20 |
|  | 2.1.21 | Debriefing . . . . . . . . . . . . . . . . . . . . . . . . . . . . . . . . . . | 20 |
| 2.2 | Study 2 | . . . . . . . . . . . . . . . . . . . . . . . . . . . . . . . . . . . . . . . . | 22 |
|  | 2.2.1 | General introduction (the *accountable* condition) . . . . . . . . . . . . . . | 22 |
|  | 2.2.2 | General introduction (the *control* condition) . . . . . . . . . . . . . . . . . | 22 |
|  | 2.2.3 | Message to participants who devoted a low level of engagement to the |  |
|  |  | main transcript task . . . . . . . . . . . . . . . . . . . . . . . . . . . . . . | 22 |
|  | 2.2.4 | Introduction (the *accountable* condition) . . . . . . . . . . . . . . . . . . . | 22 |
|  | 2.2.5 | Introduction (the *control* condition) . . . . . . . . . . . . . . . . . . . . . | 23 |
|  | 2.2.6 | Introduction (cont.; the *unjust and self-beneficial* condition) . . . . . . . . | 23 |
|  | 2.2.7 | Introduction (cont.; the *just and other-beneficial* condition) . . . . . . . . . | 23 |

|  | 2.2.8 | Measure of *liking toward agents* (all conditions) . . . . . . . . . . . . . . | 23 |
| --- | --- | --- | --- |
|  | 2.2.9 | Measure of *mind of agents* (all conditions) . . . . . . . . . . . . . . . . . . | 23 |
|  | 2.2.10 | Measure of *moral character judgments of agents* (all conditions) . . . . . . | 24 |
|  | 2.2.11 | Measure of *mental effort of participants* (all conditions) . . . . . . . . . . | 24 |
|  | 2.2.12 | Attention check question (all conditions) . . . . . . . . . . . . . . . . . . | 24 |
|  | 2.2.13 | Prompt (all conditions) . . . . . . . . . . . . . . . . . . . . . . . . . . . . | 24 |
|  | 2.2.14 | Measure of *satisfaction of agents’ decisions* (all conditions) . . . . . . . . | 24 |
|  | 2.2.15 | Measure of *fairness of agents’ decisions* (all conditions) . . . . . . . . . . | 24 |
|  | 2.2.16 | Measure of *intention of agents’ decisions* (all conditions) . . . . . . . . . . | 24 |
|  | 2.2.17 | Measure of *recalls of the mistake rates* (all conditions) . . . . . . . . . . . | 24 |
|  | 2.2.18 | Measure of *written justification* in an open-ended question (all conditions) . | 24 |
|  | 2.2.19 | Measure of *purpose of the study* in an open-ended question (all conditions) | 25 |
|  | 2.2.20 | Measure of *suspicions of the mistake rates* (all conditions) . . . . . . . . . | 25 |
|  | 2.2.21 | Debriefing . . . . . . . . . . . . . . . . . . . . . . . . . . . . . . . . . . | 25 |
| 2.3 | Study 3 | . . . . . . . . . . . . . . . . . . . . . . . . . . . . . . . . . . . . . . . . | 26 |
|  | 2.3.1 | General introduction (the *accountable* condition) . . . . . . . . . . . . . . | 26 |
|  | 2.3.2 | General introduction (the *control* condition) . . . . . . . . . . . . . . . . . | 27 |
|  | 2.3.3 | Consent (the *accountable* condition) . . . . . . . . . . . . . . . . . . . . . | 29 |
|  | 2.3.4 | Consent (the *control* condition) . . . . . . . . . . . . . . . . . . . . . . . | 29 |
|  | 2.3.5 | Introduction (the *accountable* condition) . . . . . . . . . . . . . . . . . . . | 30 |
|  | 2.3.6 | Introduction (the *control* condition) . . . . . . . . . . . . . . . . . . . . . | 31 |
|  | 2.3.7 | Prompt (all conditions) . . . . . . . . . . . . . . . . . . . . . . . . . . . . | 31 |
|  | 2.3.8 | Measure of *liking toward agents* (all conditions) . . . . . . . . . . . . . . | 31 |
|  | 2.3.9 | Measure of *harm of agents’ acts* (all conditions) . . . . . . . . . . . . . . | 31 |

|  | 2.3.10 | Measure of *moral character judgments of agents* (all conditions) . . . . . . | 31 |
| --- | --- | --- | --- |
|  | 2.3.11 | Manipulation check question (the *accountable* condition) . . . . . . . . . . | 31 |
|  | 2.3.12 | Measure of *mental effort of participants* (all conditions) . . . . . . . . . . | 36 |
|  | 2.3.13 | Debriefing . . . . . . . . . . . . . . . . . . . . . . . . . . . . . . . . . . | 36 |
| 2.4 | Study 4 | . . . . . . . . . . . . . . . . . . . . . . . . . . . . . . . . . . . . . . . . | 37 |
|  | 2.4.1 | General introduction (the *accountable - norms of justice* condition) . . . . | 37 |
|  | 2.4.2 | General introduction (the *accountable* condition) . . . . . . . . . . . . . . | 38 |
|  | 2.4.3 | Consent (all conditions) . . . . . . . . . . . . . . . . . . . . . . . . . . . | 39 |
|  | 2.4.4 | Introduction (the *accountable - norms of justice* condition) . . . . . . . . . | 40 |
|  | 2.4.5 | Introduction (the *accountable - control* condition) . . . . . . . . . . . . . . | 40 |
|  | 2.4.6 | Prompt (all conditions) . . . . . . . . . . . . . . . . . . . . . . . . . . . . | 41 |
|  | 2.4.7 | Measure of *liking toward the agent* (all conditions) . . . . . . . . . . . . . | 41 |
|  | 2.4.8 | Measure of *harm of the agents’ act* (all conditions) . . . . . . . . . . . . . | 41 |
|  | 2.4.9 | Measure of *moral character judgments of the agent* (all conditions) . . . . | 41 |
|  | 2.4.10 | Manipulation check question (the *accountable - norms of justice* condition) | 42 |
|  | 2.4.11 | Manipulation check question (the *accountable - control* condition) . . . . . | 42 |
|  | 2.4.12 | Debriefing . . . . . . . . . . . . . . . . . . . . . . . . . . . . . . . . . . | 42 |
| 2.5 | Study 5 | . . . . . . . . . . . . . . . . . . . . . . . . . . . . . . . . . . . . . . . . | 44 |
|  | 2.5.1 | General introduction (the *accountability - process* and *accountability - out-* |  |
|  |  | *come* conditions) . . . . . . . . . . . . . . . . . . . . . . . . . . . . . . . | 44 |
|  | 2.5.2 | General introduction (the *accountability - control* condition) . . . . . . . . | 44 |
|  | 2.5.3 | Consent (the *accountability - process* and *accountability - outcome* condi- |  |
|  |  | tions) . . . . . . . . . . . . . . . . . . . . . . . . . . . . . . . . . . . . . | 44 |
|  | 2.5.4 | Consent (the *accountability - control* condition) . . . . . . . . . . . . . . . | 45 |

2.5.5 Introduction (the accountability - process condition)	45
2.5.6 Introduction (the accountability - outcome condition)	45
2.5.7 Introduction (the Education - yes condition)	45
2.5.8 Introduction (all conditions)	46
2.5.9 Manipulation check questions (the *education* condition)	46
2.5.10 Prompt (all conditions)	46
2.5.11 Measure of liking toward the agent (all conditions)	47
2.5.12 Measure of harm of the agents’ act (all conditions)	47
2.5.13 Measure of moral character judgments of the agent (all conditions)	47
2.5.14 Accountability manipulation check question (all conditions)	47
2.5.15 Debriefing	47

2.6	Vignettes and check questions used in Study 3, Study 4, and Study 5	48

Bibliography	50

# List of Tables

1 Measure of *liking toward agents* (all conditions).	13
2 Measure of *mind of agents* (all conditions).	15
3 Measure of *moral character judgments* (all conditions).	17
4 Attention check question (all conditions).	18
5 Measure of *satisfaction of agents’ decisions* (all conditions).	18
6 Measure of *fairness of agents’ decisions* (all conditions).	18
7 Measure of *intention of agents’ decisions* (all conditions).	19
8 Measure of *mental effort of participants* (all conditions).	19
9 Measure of *suspicions of the mistake rates* (all conditions).	21
10 Measure of *liking toward agents* (all conditions).	32
11 Measure of *harm of agents’ acts* (all conditions).	33
12 Measure of *moral character judgments of agents* (all conditions).	34
13 Manipulation check question (the *accountable* condition).	35
14 Measure of *mental effort of participants* (all conditions).	35
15 Manipulation check question (the *accountable - norms of justice* condition).	43
16 Manipulation check question (the *accountable - control* condition).	43

# List of Figures

1 Overview of the present studies.	2

# 1. Method

1.1. *Overview of the present studies*

Figure 1 shows an overview of the present studies, including manipulations and measures.

1.2. *Exploratory variables*

In addition to our main dependent variable of interest, that is, moral character judgments, we also measured a number of exploratory variables that previous research has identified as determi- nants of the self-interest bias (Bocian et al., 2018), including measures as follows.

**Liking toward agents** in Study 1 and Study 2 was measured by two items: “I like this decider” and “I would like to interact with this decider in the future” (*α*_Study_ _1_ = .88, *M* = 3.8, *SD* = 1.7; *α*_Study_ _2_ = .89, *M* = 3.9, *SD* = 1.7). In a similar vein, in Study 3, Study 4, and Study 5, it was measured by two items: “I like Kate” and “I would like to meet Kate in the future” (*α*_Study_ _3_ =

.88, *M* = 3.3, *SD* = 1.4; *α*_Study_ _4_ = .90, *M* = 4.0, *SD* = 1.6; *α*_Study_ _5_ = .91, *M* = 3.4, *SD* = 1.4).

Participants responded to a 7-point scale ranging from 1 = *Not at all*/*Strongly Disagree* to 7 =

*Extremely*/*Strongly Agree*, with 4 representing a neutral rating in these dimensions.

**Mind of agents** were measured by six items: “This decider can communicate with others”, “... is capable of thinking”, “... plans his/her actions”, “... is sensitive to pain”, “... can experience happiness”, and “... can experience fear” (*α*_Study_ _1_ = .86, *M* = 4.8, *SD* = 1.3; *α*_Study_ _2_ = .87, *M* = 4.7, *SD* = 1.3), as presented with the same scale above.

**Satisfaction of agents’ decisions** was measured by one item: “How satisfied are you with the outcome of the decision?”, as presented with the same scale above.

**Fairness of agents’ decisions** was measured by one item: “How fair was the decision?”, as presented with the same scale above.

Study 5

2

Overview

Figure 1: Overview of the present studies.

This study explored whether

*education* has an impact on reducing the effect of the self-interest bias on moral character judgments of agents.

- Participants were randomly assigned to a condition and instructed to complete a transcript task.
- They and another passive recipient were then appointed a unanimous mistake rate for each as their performance of the task.
- An agent made either a relatively just or unjust decision that either the participant or recipient was salutary dependent on the condition.

Study 1

This study explored whether *accountability* in written form has an impact on re- ducing the effect of the self- interest bias on moral char- acter judgments of agents.

- Participants completed the same task as those from Study 1.
- These in the *accountable* condition were informed to be interrogated in writing about the reasons for their moral judgments of agents.
- These in the *control* condition were informed that their judgments would be treated completely anonymous and confidential.

Study 2

This study explored whether *accountability* in audio form has an impact on reduc-

ing the effect of the self- interest bias on moral char- acter judgments of agents.

- Participants read two moral vignettes concerning immoral acts of agents.
- The agent in each vignette is supposed to conduct a morally dubious behavior that either the participant or a passive recipient is salutary dependent on the condition.
- These in the *accountable* condition were informed to be interviewed online by the experimenter about the reasons for their ...

Study 3

This study explored whether *accountability* in audio form has an impact on reduc-

ing the effect of the self- interest bias on moral char- acter judgments of agents.

- Participants read one moral vignette concerning an immoral act of an agent.
- These in the *accountable to norms of justice* condition were informed to be interviewed online by the experimenter about the reasons for their moral judgments of the agent according to social and ethical norms such as norms of justice.
- These in the only

*accountable* condition ...

Study 4

This study explored whether *education* and process or outcome *accountability* in audio form have a joint im- pact on reducing the effect of the self-interest bias on moral character judgments of agents.

- Participants completed the same tasks as those from Study 3.
- The *education* manipulation was the same as in Study 1.
- These in the *process accountability* condition were informed to justify how they reached their moral judgments by describing their reasoning and the steps they took.

3

Overview (cont.)

| - These in the *outcome accountability* condition were informed to justify the accuracy or effectiveness of their moral judgments. - The manipulation of the *control accountability* condition was the same as in Study 3. | |
| --- | --- |
|  |  |
| - Self-interest - Education - Accountability   - Process   - Outcome | |
|  |  |

Figure 1: Overview of the present studies (cont.).

- Participants were primed to think that the agent and passive recipient were real participants.
- These in the *education* condition were educated about how the bias occurs and how to prevent it.
- These in the

*non-education* condition received no instructional information.

- Decision
- Education
- Decision
- Accountability

**–** Process

... moral judgments of agents.

- These in the *control* condition did not receive any information about being held accountable to their judgments to others.
- Self-interest
- Accountability

**–** Process

... *were* informed to be interviewed online by the experimenter about the reasons for their moral judgments of the agent.

- Self-interest
- Accountability

**–** Process

IV groups

4

DVs

- *Liking* toward agents
- *Mind* of agents
- *Moral character*

judgments

- *Satisfaction* of agents’ decisions
- *Fairness* of agents’ decisions
- *Intention* of agents’ decisions
- *Mental effort* of participants
- *Recalls* of the mistake rates
- *Purpose* of the study
- *Suspicions* of the mistake rate
- *Liking* toward agents
- *Mind* of agents
- *Moral character*

judgments

- *Satisfaction* of agents’ decisions
- *Fairness* of agents’ decisions
- *Intention* of agents’ decisions
- *Mental effort* of participants
- *Recalls* of the mistake rates
- Written *justification*
- *Suspicions* of the mistake rate
- *Purpose* of the study
- *Liking* toward agents
- *Harm* of agents’ acts
- *Moral character*

judgments

- *Mental effort* of participants
- *Liking* toward the agent
- *Harm* of the agent’ act
- *Moral character*

judgments

- *Liking* toward the agent
- *Harm* of the agent’ act
- *Moral character*

judgments

Figure 1: Overview of the present studies (cont.). *Note*: IV: Independent variable; DVs: Dependent variables. DVs are listed in their time order that occurred over the course of each study.

**Intention of agents’ decisions** was measured by one item: “How intentional was the deci- sion?”, as presented with the same scale above.

**Mental effort of participants** was measured via task performance along with self-reported mental effort that participants invested (Paas, 1992), as measured by one item: “Please indicate on the scale below how much mental effort you have invested in answering all questions about the decider.” Participants responded to a 9-point scale ranging from 1 = *very, very low mental effort* to 9 = *very, very high mental effort*, with 5 representing a neutral rating (i.e., *neither low nor high mental effort*) in these dimensions.

**Recalls of the mistake rates** of their own and the other participants with whom they were matched were measured by two yes-no questions: “Do you remember your mistake rate?” and “Do you remember the other participant’s mistake rate?”. If the answers were yes, they were further asked to specify these mistake rates.

**Purpose of the study** was measured by a free-text question.

**Suspicions of the mistake rate** were measured by one item: “Were you suspicious of your mistake rate of 23.4% given in the main task?” Participants responded to a 7-point scale ranging from -3 = *Definitely not* to 3 = *Definitely yes*, with 0 representing a neutral rating (i.e., *I am not sure*) in these dimensions.

**Harm of the agents’ acts** was measured by one item: “How harmful was Kate’s decision?” Participants responded to a 7-point scale ranging from 1 = *Completely Harmful* to 7 = *Completely Unharmful*, with 4 representing a neutral rating in these dimensions.

Also included was demographic information for each participant, such as **gender**, **age**, and **ethnic category**. Although these above exploratory variables are not central to the aims of this research, they present more opportunities to assess whether other factors may mediate or moderate

the relationship between self-interest and moral character judgments. In what follows, all signif-

icant main and interaction effects and post-hoc effects are described, and all comparisons that are not described are not significant, unless stated otherwise.

# 2. Materials

2.1. *Study 1*
     2.1.1. *General introduction (all conditions)*

We are interested in how people make decisions regarding others’ work.

2.1.2. *General introduction (cont.; all conditions)*

# You start with an additional £1 bonus for participating in this study.

First, we will pair you with other participants, and you will have 30 seconds to talk with each other.

Then, you will get an assigned role for this study. There are **responders** and **deciders**. After your role is established, we will ask you to complete a short task. Your goal will be to write as many words correctly as possible within a limited time.

After the task, **the Decider can deduct money from the bonuses based on the number of mistakes the responders made.** The Decider will not get any money for themselves, but they will decide how much the responders will receive. After the writing task, we will ask you questions about other participants and the decision.

# Remember, the bonus is additional money; regardless of the outcome of the task, you will get your base payment for participation.

—Page Break—

Right now, we are connecting you to other participants.

After everyone currently participating in the study is connected, we will establish the roles and connect you into trios - one decider and two responders. Afterwards, you will move onto the main task.

# Do not click away. After everyone is connected, this page will autoadvance.

You can observe the connection process below. You

Connecting participant68 Connecting participant27 Connecting participant49 Connecting participant63 Connecting participant72 Connecting participant98 Connecting participant103

—Page Break—

Everyone is connected, you can say ’hello’ to other participants!

# In 30 seconds, you will be moved to role assignment.

—Page Break—

In this study, there are two roles — Responders and Deciders.

Deciders can later make a decision regarding final bonuses for the responders based on the main task.

After your role is chosen, you will be able to move forward, and we will connect you into trios

— one decider and two responders.

Click the button to assign the role Assign the role

—Page Break—

Connecting to the Decider... The Responder You Connecting...

—Page Break—

Your assigned role is The Responder.

Your main task will be to transcribe as many words as possible while making as little errors as possible. Each word will disappear after you press spacebar. If the sentence changes, do not finish the sentence that disappeared, keep writing new sentence.

Please keep in mind that this task is crucial for the later decision and hence the study. If you do not actively participate in the task, you will be removed from the study, without the additional bonus.

On the next page, you will see a practice round, lasting 30 seconds, where you can get familiar with the task.

—Page Break—

This is a practice round!

Type the sentence in the box below:

Nora never knits near noisy neighbors

—Page Break—

Your mistake rate for the practice round is:

Your mistake rate is the percentage of the words that you typed incorrectly, meaning that the lower the mistake rate, the better your performance.

After the main task, the Decider will make a decision regarding your bonus based on your mistake rate.

If you are ready, go to the next page where you will proceed to the main task. The main task will last 1 minute.

—Page Break—

Type the sentence in the box below:

She sells sea shells by the sea shore

2.1.3. *Message to participants who devoted a low level of engagement to the main transcript task*

Thank you for participating in our study. We appreciate your time and effort.

Due to the nature of our research, we require engagement and thus a minimum level of accuracy for the data to be useful in our analysis.

As a result, we regret to inform you that we will not be able to continue with your participation in this survey due to your low level of engagement detected by the survey system.

Thank you again for your interest.

2.1.4. *Introduction (the* education *condition)*

# Your mistake rate is: 23.4%

When you’re ready, move to the next page, where **the Decider** will decide about the penalty based on your mistake rate. Then, we will ask you some questions about **the Decider.**

# IMPORTANT

**An instruction on how to prevent errors in moral judgments**

We use heuristics everyday when making judgments; that is, we use unconscious reasoning strategies to reach a decision quickly. Those heuristics usually work fine, but sometimes they are incorrect and lead to mistakes that creep into our thinking. Those mistakes are usually called biases.

One common bias in moral judgments is the **self-interest bias**. It refers to the unconscious tendency of people to judge others more leniently when the latter’s actions are in ways that are more beneficial for them. In other words, we pay more attention to interests that benefit us than to whether the actions made by others are in line with or violate any normative rules (e.g., fairness).

But how can you prevent the risk of self-interest bias? This can be done by applying the following thinking strategy: **Even if the person you judge has or has not benefited you, make sure your decisions about their moral characters do not depend on your interests but follow normative rules (e.g., fairness) instead.**

—Page Break—

Your mistake rate: **23.4%**

Participant27’s mistake rate: **12.1%**

Wait for the Decider to make a decision about the penalty. After the decision is made, you will be able to move forward.

Waiting for the decision... You

£1

Participant27

£1

2.1.5. *Introduction (the* non-education *condition)*

# Your mistake rate is: 23.4%

When you’re ready, move to the next page, where **the Decider** will decide about the penalty based on your mistake rate. Then, we will ask you some questions about **the Decider.**

—Page Break—

Your mistake rate: **23.4%**

Participant27’s mistake rate: **12.1%**

Wait for the Decider to make a decision about the penalty. After the decision is made, you will be able to move forward.

Waiting for the decision... You

£1

Participant27

£1

2.1.6. *Introduction (cont.; the* unjust and self-beneficial *condition)*

Based on the decision made by the **Decider**, you **lost £0.25** from your bonus, your current bonus is:

# £0.75

Now, we want to ask you questions about your perceptions of the task and the Decider.

On the following pages, you will see different statements about the **participant who decided about the bonus penalty - the Decider.**

Please rate how much you agree with each statement on a scale ranging from 1 (I strongly disagree) to 7 (I strongly agree).

2.1.7. *Introduction (cont.; the* just and other-beneficial *condition)*

Based on the decision made by the **Decider**, you **lost £0.75** from your bonus, your current bonus is:

# £0.25

Now, we want to ask you questions about your perceptions of the task and the Decider.

On the following pages, you will see different statements about the **participant who decided about the bonus penalty - the Decider.**

Please rate how much you agree with each statement on a scale ranging from 1 (I strongly disagree) to 7 (I strongly agree).

2.1.8. *Manipulation check questions (the* education *condition)*

Please indicate if the following statement is TRUE or FALSE.

The self-interest bias is a tendency to judge people as more moral when their action benefits us, albeit violates a norm, such as fairness, and less moral if the same action benefits someone else.

TRUE FALSE

—Page Break—

Please indicate if the following statement is TRUE or FALSE.

To avoid the self-interest bias, our judgments on people should depend on whether their actions are against any norm, such as fairness, rather than our personal interests.

TRUE FALSE

2.1.9. *Measure of* liking toward agents *(all conditions)*

See Table 1.

Table 1: Measure of *liking toward agents* (all conditions).

# I like this decider

1 - Strongly disagree	2	3	4	5	6	7 - Strongly agree

—Page Break

# I would like to interact with this decider in the future

1 - Strongly disagree	2	3	4	5	6	7 - Strongly agree

2.1.10. *Measure of* mind of agents *(all conditions)*

See Table 2.

2.1.11. *Measure of* moral character judgments of agents *(all conditions)*

See Table 3.

2.1.12. *Attention check question (all conditions)*

See Table 4.

2.1.13. *Prompt (all conditions)*

Now, we will ask you a few questions about **the decision** that was made about the money that was deducted from your bonus.

2.1.14. *Measure of* satisfaction of agents’ decisions *(all conditions)*

See Table 5.

2.1.15. *Measure of* fairness of agents’ decisions *(all conditions)*

See Table 6.

2.1.16. *Measure of* intention of agents’ decisions *(all conditions)*

See Table 7.

2.1.17. *Measure of* mental effort of participants *(all conditions)*

See Table 8.

Table 2: Measure of *mind of agents* (all conditions).

# This decider can communicate with others

1 - Strongly disagree	2	3	4	5	6	7 - Strongly agree

—Page Break

# This decider is capable of thinking

1 - Strongly disagree	2	3	4	5	6	7 - Strongly agree

—Page Break

# This decider plans his/her actions

1 - Strongly disagree	2	3	4	5	6	7 - Strongly agree

—Page Break

# This decider is sensitive to pain

1 - Strongly disagree	2	3	4	5	6	7 - Strongly agree

—Page Break

# This decider can experience happiness

1 - Strongly disagree	2	3	4	5	6	7 - Strongly agree

Table 2: Measure of *mind of agents* (all conditions). (continued)

—Page Break

# This decider can experience fear

1 - Strongly disagree	2	3	4	5	6	7 - Strongly agree

Table 3: Measure of *moral character judgments* (all conditions).

# This decider is just

1 - Strongly disagree	2	3	4	5	6	7 - Strongly agree

—Page Break

# This decider is moral

1 - Strongly disagree	2	3	4	5	6	7 - Strongly agree

—Page Break

# This is a trustworthy decider

1 - Strongly disagree	2	3	4	5	6	7 - Strongly agree

—Page Break

# This decider is reliable

1 - Strongly disagree	2	3	4	5	6	7 - Strongly agree

—Page Break

# This decider is honest

1 - Strongly disagree	2	3	4	5	6	7 - Strongly agree

Table 4: Attention check question (all conditions).

# If you are reading this, pick six

1 - Strongly disagree	2	3	4	5	6	7 - Strongly agree

Table 5: Measure of *satisfaction of agents’ decisions* (all conditions).

# How satisfied are you with the outcome of the decision?

1 - Not at all	2	3	4	5	6	7 - Extremely

Table 6: Measure of *fairness of agents’ decisions* (all condi- tions).

# How fair was the decision?

1 - Not at all	2	3	4	5	6	7 - Extremely

Table 7: Measure of *intention of agents’ decisions* (all con- ditions).

# How intentional was the decision?

1 - Not at all	2	3	4	5	6	7 - Extremely

Table 8: Measure of *mental effort of participants* (all conditions).

# Please indicate on the scale below how much mental effort you have invested in answering all questions about the decider.

very, very low mental effort

very	low mental effort

low mental effort

rather	low mental effort

neither	low

nor	high mental effort

rather high mental effort

high mental effort

very high mental effort

very, very high mental effort

19

2.1.18. *Measure of* recalls of the mistake rates *(all conditions)*

Do you remember your mistake rate? yes no

What was your mistake rate?

—Page Break—

Do you remember the other participant’s mistake rate? yes no

2.1.19. *Measure of* purpose of the study *(all conditions)*

Can you guess what was the real purpose of the study? Enter your observations below.

2.1.20. *Measure of* suspicions of the mistake rates *(all conditions)*

See Table 9.

2.1.21. *Debriefing*

# Thank you very much for your participation in this research. We want to provide further information about the purpose of the study and what we expect to find.

The main research question in this study was whether education could be an instructional strat- egy to change our perception of the moral character of others who made unjust decisions on behalf of our interests as compared to those who made just decisions neither on behalf of us nor the other’s interests.

Previous studies have shown that, on the one hand, our self-interest biases our moral judgments, making them more lenient when the outcome is in our favour. In other words, people judge a decider better if they get more money, even if the decisions are unfair. On the other hand, educating

people on how to prevent biases alters their decision-making and judgments by the cued principle.

Table 9: Measure of *suspicions of the mistake rates* (all conditions).

# Were you suspicious of your mistake rate of 23.4% given in the main task?

Definitely not	-2	-1	I am not sure	1	2	Definitely yes

In this study, we wanted to investigate whether educating people that self-interest biases moral character judgments (e.g., you should not use your self-interest as a source of judgment but instead of whether the decision is just or unjust) attenuates the bias.

In the study, the responder made nearly half fewer mistakes (12.1%) than you made (23.4%). To meet the aim of the study, your mistake rate was fixed and did not resemble your actual mistake rate. Depending on the research condition, the responder either got a bigger penalty (£0.75) than you got (£0.25), which was an unfair decision but beneficial for you, or both of your penalties were impartial—that is, you lost £0.75, and the responder lost £0.25, which was a fair decision. Since we manipulated the outcome of the decision, all participants will receive a bonus payment of £0.75.

Moreover, you were either provided with text-based instruction on how to prevent self-interest biases or not. In sum, we infer that the beneficial, unfair decision will make you more inclined to reveal self-interest biases than the impartial, fair decision. However, we assumed that educating people on how self-interest biases moral judgments would help them render more objective and less biased judgments when decisions were unfair but beneficial to them.

To complete this survey and receive your payment, click the arrow at the bottom of this page.

2.2. *Study 2*
     2.2.1. *General introduction (the* accountable *condition)*

# We are interested in how people make judgments regarding others’ work. Thus, at the end of the study, we will ask you to explain your impressions of the people who will participate with you, such that we can better understand what kind of information people will use to form opinions about other people.

[The rest were the same as those used in 2.1.2 General introduction (cont.; all conditions).]

2.2.2. *General introduction (the* control *condition)*

textbfWe are interested in how people make judgments regarding others’ work. Your judgments will be completely anonymous and confidential. We will not know your responses because they will be first aggregated and then analyzed.

[The rest were the same as those used in 2.1.2 General introduction (cont.; all conditions).]

2.2.3. *Message to participants who devoted a low level of engagement to the main transcript task*

See 2.1.3 Message to participants who devoted a low level of engagement to the main transcript task.

2.2.4. *Introduction (the* accountable *condition)*

Based on the decision made by the **Decider**, you **lost £0.25** from your bonus, your current bonus is:

# £0.75

Now, we want to ask you questions about your perceptions of the task and the Decider.

On the following pages, you will see different statements about the **participant who decided about the bonus penalty - the Decider.**

Please rate how much you agree with each statement on a scale ranging from 1 (I strongly disagree) to 7 (I strongly agree).

# REMINDER

**We will ask you to provide a written justification for your judgments at the end of the** **study.**

2.2.5. *Introduction (the* control *condition)*

Based on the decision made by the **Decider**, you **lost £0.25** from your bonus, your current bonus is:

# £0.75

Now, we want to ask you questions about your perceptions of the task and the Decider.

On the following pages, you will see different statements about the **participant who decided about the bonus penalty - the Decider.**

Please rate how much you agree with each statement on a scale ranging from 1 (I strongly disagree) to 7 (I strongly agree).

2.2.6. *Introduction (cont.; the* unjust and self-beneficial *condition)*

See 2.1.6 Introduction (cont.; the *unjust and self-beneficial* condition).

2.2.7. *Introduction (cont.; the* just and other-beneficial *condition)*

See 2.1.7 Introduction (cont.; the *just and other-beneficial* condition).

2.2.8. *Measure of* liking toward agents *(all conditions)*

See Table 1.

2.2.9. *Measure of* mind of agents *(all conditions)*

See Table 2.

2.2.10. *Measure of* moral character judgments of agents *(all conditions)*

See Table 3.

2.2.11. *Measure of* mental effort of participants *(all conditions)*

See Table 8.

2.2.12. *Attention check question (all conditions)*

See Table 4.

2.2.13. *Prompt (all conditions)*

See 2.1.13 Prompt (all conditions).

2.2.14. *Measure of* satisfaction of agents’ decisions *(all conditions)*

See Table 5.

2.2.15. *Measure of* fairness of agents’ decisions *(all conditions)*

See Table 6.

2.2.16. *Measure of* intention of agents’ decisions *(all conditions)*

See Table 7.

2.2.17. *Measure of* recalls of the mistake rates *(all conditions)*

See 2.1.18 Measure of *recalls of the mistake rates* (all conditions).

2.2.18. *Measure of* written justification *in an open-ended question (all conditions)*

Please proceed to the next screen where you can justify your judgments of the Decider in written form.

Please note that you have 60 seconds to do so.

—Page Break—

Please justify the reasons behind your judgments of the Decider.

2.2.19. *Measure of* purpose of the study *in an open-ended question (all conditions)*

See 2.1.19 Measure of *purpose of the study* (all conditions).

2.2.20. *Measure of* suspicions of the mistake rates *(all conditions)*

See Table 9.

2.2.21. *Debriefing*

# Thank you very much for your participation in this research. We want to provide further information about the purpose of the study and what we expect to find.

The main research question in this study was whether *accountability*—the expectation by indi- viduals that they may need to justify their judgments in written form— could contextually change their perceptions of the moral character of an agent who made *unjust* decisions on behalf of our interests as compared to an agent who made *just* decisions neither on behalf of our interests nor on behalf of a passive recipient’s interests.

Previous studies have shown that, on the one hand, our self-interest biases our moral judgments, making them more lenient when the outcome is in our favour—an anomaly termed the self-interest bias. In other words, people judge a decider better if they get more money, even though the deci- sions are unfair. On the other hand, accountability has been found to lead to less biased decisions due to higher cognitive effort. In this study, we wanted to investigate whether people who are held accountable for their judgments could be expected to be motivated to present themselves as good decision makers, thus attenuating the self-interest bias.

More precisely, the responder made nearly half fewer mistakes (12.1%) than you made (23.4%).

To meet the aim of the study, your mistake rate was fixed and did not resemble your actual mis- take rate. Besides, depending on the research condition, the responder either got a bigger penalty (£0.75) than you got (£0.25), which was an unfair decision but was beneficial for you, or both of your penalties were impartial—that is, you lost £0.75, and the responder lost £0.25, which was rather a fair decision. Moreover, you were either requested to justify the reasons behind your judg- ments or were not requested to do so. Nevertheless, all participants will receive a unanimous bonus payment of £0.75.

In sum, we infer that the beneficial, unfair decision will make you more inclined to reveal the self-interest bias than the impartial, fair decision. However, we assumed that people who are requested to be accountable for their judgments would render more objective and less biased judgments when decisions were unfair but beneficial to them than those who who are not requested to do so.

To complete this survey and receive your payment, click the arrow at the bottom of this page.

2.3. *Study 3*
     2.3.1. *General introduction (the* accountable *condition)*

# Social Impressions of Others

**Aims of the Study:**

We would like to invite you to participate in a study investigating how people form impressions of others based on the information provided about the latter’s behaviors.

# Eligibility Requirements:

To take part in the study,

- You should be at least 18 years old;
- You should be fluent in English.

# What you will need to do and your time commitment:

After consenting to participate in this study, you will be presented with two short scenarios occurred in certain social contexts, in each of which you, a target person, and a third person are presumably involved. Your tasks are, in short, as follows:

- First, you will need to read each scenario carefully as if you were part of it.
- Next, you will need to answer several questions about your impressions of the target person, who behaved in different ways in the different contexts.
- Last, you will need to justify the **reasons for your judgments**. In doing so, an experimenter will invite you to join in an audio interview.

The study may take around 8 mins.

# Risks/Discomforts involved in participating this study:

We do not anticipate any risk or discomfort.

# Confidentiality of your data:

Any responses you provide will be treated confidentially. Any publication resulting from this work will report only aggregated findings or fully anonymized examples that will not identify your personal data. However, we will need you to identify your Prolific ID in case of data withdrawal.

2.3.2. *General introduction (the* control *condition)*

# Social Impressions of Others

**Aims of the Study:**

We would like to invite you to participate in a study investigating how people form impressions of others based on the information provided about the latter’s behaviors.

# Eligibility Requirements:

To take part in the study,

- You should be at least 18 years old;
- You should be fluent in English.

# What you will need to do and your time commitment:

After consenting to participate in this study, you will be presented with two short scenarios occurred in certain social contexts, in each of which you, a target person, and a third person are presumably involved. Your tasks are, in short, as follows:

- First, you will need to read each scenario carefully as if you were part of it.
- Last, you will need to answer several questions about your impressions of the target person, who behaved in different ways in the different contexts.

The study may take around 8 mins.

# Risks/Discomforts involved in participating this study:

We do not anticipate any risk or discomfort.

# Confidentiality of your data:

Any responses you provide will be treated confidentially. Any publication resulting from this work will report only aggregated findings or fully anonymized examples that will not identify your personal data. However, we will need you to identify your Prolific ID in case of data withdrawal.

2.3.3. *Consent (the* accountable *condition)*

Please read the following statements and confirm if you agree with them:

- I understand that my participation is voluntary and that I am free to withdraw at any time without giving any reason.
- I understand that the experimenter will audibly interview me about the reasons for my judg- ments at the end of this study. For analysis purposes only, the interview will be recorded.
- I understand that my data including the audio interview will be treated confidentially, and any publication resulting from this work will report only data that does not identify me nor my voice.
- I understand that my responses including the audio interview can only be traceable to the researchers in this study. My responses except for the audio interview, however, may only be anonymously shared with other researchers or made available in online data repositories.
- I freely agree to participate in this study.
2.3.4. *Consent (the* control *condition)*

Please read the following statements and confirm if you agree with them:

- I understand that my participation is voluntary and that I am free to withdraw at any time without giving any reason.
- I understand that my data will be treated confidentially, and any publication resulting from this work will report only data that does not identify me. My anonymized responses, how- ever, may only be anonymously shared with other researchers or made available in online data repositories.
- I freely agree to participate in this study.
2.3.5. *Introduction (the* accountable *condition)*

There are no right or wrong answers to the questions we ask. We are only interested in your impressions of others. Please do however think carefully before indicating your judgments, since the answers you provide are important and may help understand impression formation in social cognition.

Please note that once you are done filling in and submitting all the questions, you will be invited to an audio interview, in which an experimenter will interview you in detail about the **reasons for your judgments**. This interview is meant to assess the reasoning that has led you to make each single decision.

In this study, you will read two short scenarios, each of which describes an interaction between you and a target person, alongside a third person, that is supposedly occurred in a social context, followed by a number of questions that address to ask your impressions of this target person, including their potential character traits.

For each scenario, please read it carefully as we will check your comprehension of it by asking a question about the target person’s action that is portrayed in the scenario. Note that you will have as much time as you want but no less than 15 seconds to read each scenario.

2.3.6. *Introduction (the* control *condition)*

There are no right or wrong answers to the questions we ask. We are only interested in your impressions of others.

In this study, you will read two short scenarios, each of which describes an interaction between you and a target person, alongside a third person, that is supposedly occurred in a social context, followed by a number of questions that address to ask your impressions of this target person, including their potential character traits.

For each scenario, please read it carefully as we will check your comprehension of it by asking a question about the target person’s action that is portrayed in the scenario. Note that you will have as much time as you want but no less than 15 seconds to read each scenario.

2.3.7. *Prompt (all conditions)*

Now, please direct your attention to the following scenario.

2.3.8. *Measure of* liking toward agents *(all conditions)*

See Table 10.

2.3.9. *Measure of* harm of agents’ acts *(all conditions)*

See Table 11.

2.3.10. *Measure of* moral character judgments of agents *(all conditions)*

See Table 12.

2.3.11. *Manipulation check question (the* accountable *condition)*

See Table 13.

Table 10: Measure of *liking toward agents* (all conditions).

1 - Strongly Disagree

2 - Disagree
3 - Somewhat Disagree
4 - Neutral
5 - Somewhat Agree
6 - Agree
7 - Strongly Agree

32

# I like Kate

**I would like to meet Kate in the future**

Table 11: Measure of *harm of agents’ acts* (all conditions).

# How harmful was Kate’s decision?

1 - Completely Harmful
2 - Mostly Harm- ful
3 -	Somewhat Harmful
4 - Neutral
5 -	Somewhat Unharmful
6 - Mostly Un- harmful
7 - Completely Unharmful

33

Table 12: Measure of *moral character judgments of agents* (all conditions).

1 - Strongly Disagree

2 - Disagree
3 - Somewhat Disagree
4 - Neutral
5 - Somewhat Agree
6 - Agree
7 - Strongly Agree

34

**Kate is fair**

**Kate is moral**

**Kate is trustworthy**

**Kate is reliable**

**Kate is honest**

Table 13: Manipulation check question (the *accountable* condition).

# Do you expect to explain the reasons for your judgments to an experimenter?

1 - Definitely Not	2 - Not	3 - Slightly Not	4 - I am not sure	5 - SlightlyYes	6 - Yes	7 - Definitely Yes

Table 14: Measure of *mental effort of participants* (all conditions).

# Please indicate how much mental effort you have invested in answering all the questions above.

Very,	Very Low

Very Low	Low	Rather Low

Neither	Low Nor High

Rather High

High

Very High

Very,	Very High

35

2.3.12. *Measure of* mental effort of participants *(all conditions)*

See Table 14.

2.3.13. *Debriefing*

Thank you very much for participating in this study. Here below we provide further information about the purpose and hypotheses of the study.

We aimed to investigate whether accountability—the explicit expectation by people that they may need to justify their judgments—could contextually change people’s perceptions of moral characters of others for justice breaches. Previous studies have shown that, on the one hand, our self-interest biases and makes our moral judgments more lenient when the outcome is in our favor—an anomaly termed the self-interest bias. In other words, people judge others more morally when the former benefits from the latter’s behaviors compared to not benefiting, even though those behaviors are morally dubious or unjust.

On the other hand, accountability is a powerful debiasing mechanism. In other words, holding people accountable to their judgments may attenuate biased judgments due to raised systematic judgment processes and responsibility appraisals rather than heuristic thinking. Thus, we hypoth- esized that accountability may attenuate the self-interest bias.

Note that you might be requested to join in an audio interview to provide oral justifications for your judgments. However, this *ad hoc* interview is NOT necessary anymore since the effect of ac- countability is expected to be occurred already during the process of your judgments, if applicable. Nor would any nondeceptive commission be suitable for investigating accountability in this study.

Thank you for your participation in our study.

2.4. *Study 4*
     2.4.1. *General introduction (the* accountable - norms of justice *condition)*

# Social Impressions of Others

**Aims of the Study:**

We would like to invite you to participate in a study investigating how people form impressions of others based on the information provided about the others’ behaviors.

# Eligibility Requirements:

To take part in the study,

- You should be at least 18 years old;
- You should be fluent in English.

# What you will need to do and your time commitment:

After consenting to participate in this study, you will be presented with one short scenario occurred in a certain social context, in which you, a target person, and a third person are presumed to be involved. Your tasks are, in short, as follows:

- First, you will need to read this scenario carefully as if you were part of it.
- Next, you will need to answer several questions about your impressions of the target person.
- Last, you will need to justify the **reasons for your judgments according to social and ethical norms such as norms of justice**. In doing so, an experimenter will invite you to join in **an audio interview**.

The study may take around 5 mins.

# Risks/Discomforts involved in participating this study:

We do not anticipate any risk or discomfort.

# Confidentiality of your data:

Any responses you provide will be treated confidentially. Any publication resulting from this work will report only aggregated findings or fully anonymized examples that will not identify your personal data. However, we will need you to identify your Prolific ID in case of data withdrawal.

2.4.2. *General introduction (the* accountable *condition)*

# Social Impressions of Others

**Aims of the Study:**

We would like to invite you to participate in a study investigating how people form impressions of others based on the information provided about the others’ behaviors.

# Eligibility Requirements:

To take part in the study,

- You should be at least 18 years old;
- You should be fluent in English.

# What you will need to do and your time commitment:

After consenting to participate in this study, you will be presented with one short scenario occurred in a certain social context, in which you, a target person, and a third person are presumed to be involved. Your tasks are, in short, as follows:

- First, you will need to read this scenario carefully as if you were part of it.
- Next, you will need to answer several questions about your impressions of the target person.
- Last, you will need to justify the **reasons for your judgments**. In doing so, an experimenter will invite you to join in **an audio interview**.

The study may take around 5 mins.

# Risks/Discomforts involved in participating this study:

We do not anticipate any risk or discomfort.

# Confidentiality of your data:

Any responses you provide will be treated confidentially. Any publication resulting from this work will report only aggregated findings or fully anonymized examples that will not identify your personal data. However, we will need you to identify your Prolific ID in case of data withdrawal.

2.4.3. *Consent (all conditions)*

Please read the following statements and confirm if you agree with them:

- I understand that my participation is voluntary and that I am free to withdraw at any time without giving any reason.
- I understand that the experimenter will audibly interview me about the reasons for my judg- ments at the end of this study. For analysis purposes only, the interview will be recorded.
- I understand that my data including **the audio interview** will be treated confidentially, and

any publication resulting from this work will report only data that does not identify me nor my voice.

- I understand that my responses including **the audio interview** can only be traceable to the researchers in this study. My responses except for the audio interview, however, may only be anonymously shared with other researchers or made available in online data repositories.
- I freely agree to participate in this study.
2.4.4. *Introduction (the* accountable - norms of justice *condition)*

There are no right or wrong answers to the questions we ask. We are only interested in your impressions of others. Please do however think carefully before indicating your judgments, since the answers you provide are important and may help understand impression formation in social cognition.

Please note that once you are done filling in and submitting all the questions, you will be invited to **an audio interview**, in which an experimenter will interview you in detail about the **reasons for your judgments according to social and ethical norms such as norms of justice**. This interview is meant to assess the reasoning that has led you to make each single decision.

In this study, you will read one short scenario, which describes an interaction between you and a target person, alongside a third person, that is supposed to be occurred in a social context, followed by a number of questions that address to ask your impressions of this target person, including their potential character traits.

For this scenario, please read it carefully as we will check your comprehension of it by asking a question about the target person’s action that is portrayed in the scenario. Note that you will have as much time as you want but no less than 15 seconds to read this scenario.

2.4.5. *Introduction (the* accountable - control *condition)*

There are no right or wrong answers to the questions we ask. We are only interested in your impressions of others. Please do however think carefully before indicating your judgments, since

the answers you provide are important and may help understand impression formation in social cognition.

Please note that once you are done filling in and submitting all the questions, you will be invited to **an audio interview**, in which an experimenter will interview you in detail about the **reasons for your judgments**. This interview is meant to assess the reasoning that has led you to make each single decision.

In this study, you will read one short scenario, which describes an interaction between you and a target person, alongside a third person, that is supposed to be occurred in a social context, followed by a number of questions that address to ask your impressions of this target person, including their potential character traits.

For this scenario, please read it carefully as we will check your comprehension of it by asking a question about the target person’s action that is portrayed in the scenario. Note that you will have as much time as you want but no less than 15 seconds to read this scenario.

2.4.6. *Prompt (all conditions)*

Now, please direct your attention to the scenario.

2.4.7. *Measure of* liking toward the agent *(all conditions)*

See Table 10.

2.4.8. *Measure of* harm of the agents’ act *(all conditions)*

See Table 11.

2.4.9. *Measure of* moral character judgments of the agent *(all conditions)*

See Table 12.

2.4.10. *Manipulation check question (the* accountable - norms of justice *condition)*

See Table 15.

2.4.11. *Manipulation check question (the* accountable - control *condition)*

See Table 16.

2.4.12. *Debriefing*

# Exploring the Effect of Accountability on the Self-Interest Bias in Moral Character Judgments of Others

Thank you very much for participating in this study. Here below we provide further information about the purpose and hypotheses of the study.

We aimed to investigate whether accountability—the explicit expectation by people that they may need to justify their judgments—could contextually change people’s perceptions of moral characters of others for justice breaches. Previous studies have shown that, on the one hand, our self-interest biases and makes our moral judgments more lenient when the outcome is in our favor—an anomaly termed the self-interest bias (Bocian & Wojciszke, 2014). In other words, people judge others more morally when the former benefits from the latter’s behaviors compared to not benefiting, even though those behaviors are morally dubious or unjust.

On the other hand, accountability is a powerful debiasing mechanism (e.g., Bersoff & Miller, 1993). In other words, holding people accountable to their judgments may attenuate biased judg- ments due to raised systematic judgment processes and responsibility appraisals rather than heuris- tic thinking. Thus, we hypothesized that holding people accountable to their judgments, especially according to social and ethical norms, may attenuate the self-interest bias.

Note that you were requested to join in an audio interview to provide oral justifications for your

Table 15: Manipulation check question (the *accountable - norms of justice* condition).

43

# Do you expect to explain the reasons for your judgments to an experimenter according to social and ethical norms such as norms of justice?

1 - Definitely Not	2 - Not	3 - Slightly Not	4 - I am not sure	5 - SlightlyYes	6 - Yes	7 - Definitely Yes

Table 16: Manipulation check question (the *accountable - control* condition).

# Do you expect to explain the reasons for your judgments to an experimenter?

1 - Definitely Not	2 - Not	3 - Slightly Not	4 - I am not sure	5 - SlightlyYes	6 - Yes	7 - Definitely Yes

judgments. However, this *ad hoc* interview is NOT necessary anymore since the effect of account- ability was expected to be occurred already during the process of your judgments, if applicable. Nor would any nondeceptive commission be suitable for investigating accountability in this study.

Thank you for your participation in our study. References:

Bersoff, D. M., & Miller, J. G. (1993). Culture, context, and the development of moral ac- countability judgments. *Developmental Psychology*, *29*, 664-676.

Bocian, K., & Wojciszke, B. (2014). Self-interest bias in moral judgments of others’ actions.

*Personality and Social Psychology Bulletin*, *40*, 898-909.

2.5. *Study 5*
     2.5.1. *General introduction (the* accountability - process *and* accountability - outcome *conditions)* [The instructions were the same as those used in 2.3.1 General introduction (the *accountable* condition), except that the sentence “The study may take around 8 mins.” was revised as follows:] The study may take around 8 minutes in total, including 5 minutes to complete answering the questions and another 3 minutes to justify your reasons in the audio interview. Nevertheless, we

encourage you to proceed at your own pace.

2.5.2. *General introduction (the* accountability - control *condition)*

[The instructions were the same as those used in 2.3.2 General introduction (the *control* condi- tion), except that the sentence “The study may take around 8 mins.” was revised as follows:]

The study may last around 8 minutes. Nevertheless, we encourage you to proceed at your own pace.

2.5.3. *Consent (the* accountability - process *and* accountability - outcome *conditions)*

See 2.3.3 Consent (the *accountable* condition).

2.5.4. *Consent (the* accountability - control *condition)*

See 2.3.4 Consent (the *control* condition).

2.5.5. *Introduction (the* accountability - process *condition)*

Please note that once you are done filling in and submitting all the questions, you will be invited to an audio interview, in which an experimenter will interview you in detail about **how you reached your moral judgments. Please be prepared to describe your reasoning and the steps you took.** This interview is meant to assess the reasoning that has led you to make each single decision.

2.5.6. *Introduction (the* accountability - outcome *condition)*

Please note that once you are done filling in and submitting all the questions, you will be invited to an audio interview, in which an experimenter will interview you in detail about **the accuracy or effectiveness of your moral judgments. The quality of your result will be compared with others.** This interview is meant to assess the reasoning that has led you to make each single decision.

2.5.7. *Introduction (the* Education - yes *condition)*

# An instruction on how to prevent errors in moral judgments

We use heuristics everyday when making judgments; that is, we use unconscious reasoning strategies to reach a decision quickly. Those heuristics usually work fine, but sometimes they are incorrect and lead to mistakes that creep into our thinking. Those mistakes are usually called biases.

One common bias in moral judgments is the **self-interest bias**. It refers to the unconscious tendency of people to judge others more leniently when the latter’s actions are in ways that are

more beneficial for them. In other words, we pay more attention to interests that benefit us than to whether the actions made by others are in line with or violate any normative rules (e.g., fairness).

But how can you prevent the risk of self-interest bias? This can be done by applying the following thinking strategy: **Even if the person you judge has or has not benefited you, make sure your decisions about their moral characters do not depend on your interests but follow** **normative rules (e.g., fairness) instead.**

2.5.8. *Introduction (all conditions)*

In this study, you will read two short scenarios, each of which describes an interaction between you and a target person, alongside a third person, that is supposedly occurred in a social context, followed by a number of questions that address to ask your impressions of this target person, including their potential character traits.

For each scenario, please read it carefully as we will check your comprehension of it by asking a question about the target person’s action that is portrayed in the scenario. Note that you will have as much time as you want but no less than 15 seconds to read each scenario.

Please note that there are no right or wrong answers to the questions we ask. We are only interested in your impressions of others. Please do however think carefully before indicating your judgments, since the answers you provide are important and may help understand impression for- mation in social cognition.

2.5.9. *Manipulation check questions (the* education *condition)*

See 2.1.8 Manipulation check questions (the *education* condition).

2.5.10. *Prompt (all conditions)*

Now, please direct your attention to the scenario.

2.5.11. *Measure of* liking toward the agent *(all conditions)*

See Table 10.

2.5.12. *Measure of* harm of the agents’ act *(all conditions)*

See Table 11.

2.5.13. *Measure of* moral character judgments of the agent *(all conditions)*

See Table 12.

2.5.14. *Accountability manipulation check question (all conditions)*

See Table 16.

2.5.15. *Debriefing*

Thank you very much for participating in this study. Here below we provide further information about the purpose and hypotheses of the study.

We aimed to investigate whether educating people on how to prevent biases and accountability— the explicit expectation by people that they may need to justify their judgments—could contextu- ally change people’s perceptions of moral characters of others for justice breaches. Previous studies have shown that, on the one hand, our self-interest biases and makes our moral judgments more lenient when the outcome is in our favor—an anomaly termed the self-interest bias. In other words, people judge others more morally when the former benefits from the latter’s behaviors compared to not benefiting, even though those behaviors are morally dubious or unjust.

On the other hand, education and accountability are powerful debiasing mechanisms. In other words, educating people on how to prevent biases alters their decision-making and judgments by the cued principle. Holding people accountable to their judgments may attenuate biased judg-

ments due to raised systematic judgment processes and responsibility appraisals rather than heuris-

tic thinking. Thus, we hypothesized that education and/or accountability may attenuate the self- interest bias.

[The rest were the same as those used in 2.3.13 Debriefing.]

2.6. *Vignettes and check questions used in Study 3, Study 4, and Study 5*

There were two self-interest versions of the vignettes, either *involved* or *control*. The involved version is shown in brackets. The moral dilemmas were narrated by the authors. After reading each vignette, participants answered a dichotomous yes-no check question.

*General instructions*. In this study, you will read two short scenarios, each of which describes an interaction between you and a target person, alongside a third person, that is supposedly oc- curred in a social context, followed by a number of questions that address to ask your impressions of this target person, including their potential character traits.

For each scenario, please read it carefully as we will check your comprehension of it by asking a question about the target person’s action that is portrayed in the scenario. Note that you will have as much time as you want but no less than 15 seconds to read each scenario.

*Vignette—Class*. Imagine that you are a student waiting for an important exam. You hear two people from your class, **Tom** and his friend **Kate** discussing a mutual acquaintance. **Tom** mentions that the acquaintance often cheats on exams. Shortly after their conversation, the door to the exam hall opens and together with **Tom**, **Kate** and other people from the class you get in. Students take seats around you with one free chair apart. The test will last 40 minutes. Shortly after the test began, you notice that **Kate**, who is sitting in front of you, deliberately looks at her friend’s paper. When the exam invigilator walks away, **Kate** takes the opportunity and starts to copy her friend’s answer sheet. After 10 minutes, you notice that **Kate** has finished copying the answers. [You poke Kate and ask if she would show you her answer sheet. **Kate** agrees and shows you her answer

sheet. You copy out the answers quickly, answering all questions.]

*Check question—Class*. If Kate copied the answers from her friend?

*Vignette—Politics*. Imagine that you are a citizen and you have an appointment to the con- stituency office to meet your local MP called **Kate**. You sit in the waiting room and you hear **Kate** discussing with her colleague, **Tom**, about state parliamentary rules. Right after the conversation with **Tom**, you heard **Kate** talking with a citizen and mediating to the local Tax Office to get the citizen’s legit fine due to illegal parking deleted from their tax records. At the end of their appoint- ment, you heard the citizen thanking **Kate** for her mediation and promising a money donation. [Right after you meet with **Kate** and ask for a political favour to mediate to the local Tax Office and get your legit fine due to your illegal parking deleted. **Kate** agrees and immediately mediates so that you will have your fine deleted from your tax records.]

*Check question—Politics*. If Kate deleted the fine for Tom?

*Vignette—Organization*. Imagine that you work in a life and fire protection company waiting for your annual review appointment with your line manager, **Kate**. You hear two people from your company, **Tom** and his friend **Mark** discussing a mutual acquaintance. **Tom** mentions that **Kate** recently offered him a promotion, although he did not meet all the objectives of his contract as he has not passed safety training. This might result in serious risks when **Tom** rescues people in danger due to a lack of the training. Due to this promotion, **Kate** received a bribe from **Tom**. Right after the conversation with **Tom**, you meet with **Kate**. [During the meeting you explain that although you did not meet all the objectives of your contract due to the above-mentioned reason, you deserve a promotion. **Kate** agreed to offer you a promotion at this stage and initiated the process of your promotion.]

*Check question—Organization*. If Kate offered a promotion to Tom?

*Vignette—Soft*. Imagine that you are in a café with **Tom** and **Kate** discussing about a mutual

acquaintance. **Tom** mentions that the acquaintance often downloads software from Internet ille- gally. After few minutes, **Kate** says that recently she has illegally downloaded a very expensive software which saved her a lot of money. [You asked **Kate**, if she could share it with you and she agrees.]

*Check question—Soft*. If Kate downloaded the costly software?

# Bibliography

Bocian, K., Baryla, W., Kulesza, W. M., Schnall, S., & Wojciszke, B. (2018). The mere liking effect: Attitudinal influences on attributions of moral character. *Journal of Experimental Social Psychology*, *79*, 9–20. doi:[10.1016/](http://dx.doi.org/10.1016/j.jesp.2018.06.007) [j.jesp.2018.06.007](http://dx.doi.org/10.1016/j.jesp.2018.06.007).

Paas, F. (1992). Training strategies for attaining transfer of problem-solving skill in statistics: A cognitive-load approach. *Journal of Educational Psychology*, *84*, 429–434. doi:[10.1037/0022-0663.84.4.429](http://dx.doi.org/10.1037/0022-0663.84.4.429).
