# Supplementary material for: Testing Education and Accountability as Moderators of the Self-Interest Bias in Moral Character Judgments
Source: Pers Soc Psychol Bull. 2025 Aug 28;52(10):3127–43. doi: 10.1177/01461672251364308 (PMC13507493; doi:10.1177/01461672251364308)
Supplement: Supplementary file 2 [file sj-docx-2-psp-10.1177_01461672251364308.docx]

# Supplementary Materials, additional measures, and analyses for:

“Testing Education and Accountability as Moderators of the Self-Interest Bias in Moral Character Judgments”

*Note*. Raw datasets and R Notebooks (statistical analyses results) for each study are available on

| the Open Science Framework at <https://osf.io/gnz7c>. |  |
| --- | --- |
| **Contents** |  |
| **Contents** | **i** |
| **List of Tables** | **iii** |
| **List of Figures** | **iv** |
| **1 Additional measures** | **1** |
| 1.1 Ethnic categories . . . . . . . . . . . . . . . . . . . . . . . . . . . . . . . . . . . | 1 |
| 1.2 Results of main analysis with the full samples . . . . . . . . . . . . . . . . . . . . | 1 |
| 1.3 Results of main analysis when excluding those participants who incorrectly an- |  |
| swered the two manipulation check questions in the education condition in Study |  |
| 1 (*n* = 24) and Study 5 (*n* = 20) . . . . . . . . . . . . . . . . . . . . . . . . . . . . | 1 |
| 1.4 The unanimous mistake rate as a covariate . . . . . . . . . . . . . . . . . . . . . . | 3 |
| 1.5 Statistical analyses of exploratory variables . . . . . . . . . . . . . . . . . . . . . | 7 |
| 1.5.1 Study 1 . . . . . . . . . . . . . . . . . . . . . . . . . . . . . . . . . . . . | 7 |
| 1.5.2 Study 2 . . . . . . . . . . . . . . . . . . . . . . . . . . . . . . . . . . . . | 11 |
| 1.5.3 Study 3 . . . . . . . . . . . . . . . . . . . . . . . . . . . . . . . . . . . . | 16 |
| 1.5.4 Study 4 . . . . . . . . . . . . . . . . . . . . . . . . . . . . . . . . . . . . | 17 |

**2**

| 1.5.5 | | Study 5 . . . . . . . . . . . . . . . . . . . . . . . . . . . . . . . . . . . . | 18 |
| --- | --- | --- | --- |
| **Variables included in the surveys of the present studies on Qualtrics** **19** | | | |
| 2.1 | Study 1 | . . . . . . . . . . . . . . . . . . . . . . . . . . . . . . . . . . . . . . . . | 19 |
| 2.2 | Study 2 | . . . . . . . . . . . . . . . . . . . . . . . . . . . . . . . . . . . . . . . . | 27 |
| 2.3 | Study 3 | . . . . . . . . . . . . . . . . . . . . . . . . . . . . . . . . . . . . . . . . | 27 |
| 2.4 | Study 4 | . . . . . . . . . . . . . . . . . . . . . . . . . . . . . . . . . . . . . . . . | 27 |
| 2.5 | Study 5 | . . . . . . . . . . . . . . . . . . . . . . . . . . . . . . . . . . . . . . . . | 34 |

**Bibliography**	**36**

**List of Tables**

1 Ethnic categories by study (%).	2
2 Variables included in the survey of Study 1 on Qualtrics.	20
3 Variables exclusively included in the survey of Study 2 on Qualtrics.	27
4 Variables included in the survey of Study 3 on Qualtrics.	28
5 Variables exclusively included in the survey of Study 4 on Qualtrics.	34
6 Variables exclusively included in the survey of Study 5 on Qualtrics.	35

**List of Figures**

1 Results from Study 1 after excluding those participants who incorrectly answered

the two manipulation check questions in the education condition (n = 24).	3

2 Results from Study 1 after excluding those participants who were definitely suspi- cious of the mistake rate given in the main task: Moral character judgments as a

function of education (no or yes).	5

3 Effect of actual mistake rates as a covariate in the main task of Study 2 on moral character judgments: Self-interest as a determiner	7
4 Inverse-document-frequency-weighted word cloud showing common single words in sizes proportional to their frequency in the response to the free-text question (on

what purpose participants thought about the study) in Study 1.	10

5 Participant distributions of the ratings of suspicions of the mistake rate in response to the question, “Were you suspicious of your mistake rate of 23.4% given in the

main task?”, in Study 1.	11

6 Path diagram showing mediation analysis in Study 1.	11
7 Inverse-document-frequency-weighted word cloud showing common single words in sizes proportional to their frequency in the response to the free-text question (on

what reasons participants justified their judgments of the agents) in Study 2.	13

8 Participant distributions of the ratings of suspicions of the mistake rate in response to the question, “Were you suspicious of your mistake rate of 23.4% given in the

main task?”, in Study 2.	14

9 Inverse-document-frequency-weighted word cloud showing common single words in sizes proportional to their frequency in the response to the free-text question (on

what purpose participants thought about the study) in Study 2.	15

10 Path diagram showing mediation analysis in Study 2.	16
11 Path diagram showing mediation analysis in Study 3.	17
12 Path diagram showing mediation analysis in Study 4.	19
13 Path diagram showing mediation analysis in Study 5.	19

# 1. Additional measures

1.1. *Ethnic categories*

Here, among the final sample of each study, we report ethnic categories (see Table 1). When interpreting this table, we observed 2, 67, 29, 34, and 28 non-responding participants across Study 1 and Study 5, respectively. We also note that 3, 4, and 5 participants preferred not to say this demographic information in the last three studies, respectively.

1.2. *Results of main analysis with the full samples*

Because our hypotheses are predicted on the assumptions that participants (1) devote their at- tentional engagement to the survey in Study 1 and Study 2, and (2) understand the agent’s action in the vignette in Study 3, Study 4, and Study 5, those who transcribed less than five any words in the practice trial in the first two studies or incorrectly answered any of the attention or vignette check questions in the last three studies were excluded from analysis for these studies. However, the out- comes of these analyses on moral character judgments generally did not depend on these exclusion criteria. When the analyses were repeated on all participants for these studies, the comparisons had similar significance levels as the primary analyses.

1.3. *Results of main analysis when excluding those participants who incorrectly answered the two manipulation check questions in the education condition in Study 1 (*n *= 24) and Study 5 (*n *= 20)*

In Study 1, a 2 (decision: unjust but self-beneficial vs. just but other-beneficial) × 2 (education: yes vs. no) analysis of variance (ANOVA) revealed a significant interaction effect (cf., Figure 1), *F*(1, 660) = 7.87, *p* = .005, *η²*_p_ = 0.012, 95% CI [-0.90, 0.82], which was consistent with our hypothesis. Pairwise comparisons showed that, in the non-education condition, those participants

Table 1: Ethnic categories by study (%).

|  | | | | American |  | Native |  |
| --- | --- | --- | --- | --- | --- | --- | --- |
|  |  |  |  | Indian or |  | Hawaiian |  |
| Study | *N* | Caucasian | African  American | Alaska  native | Asian | or Pacific  Islander | Other^a^ |
| Study 1 | 688 | 67.30 | 16.90 | 1.60 | 9.88 | 0.44 | 3.88 |
| Study 2 | 967 | 65.80 | 15.50 | 0.93 | 8.39 | 0.00 | 9.38 |
| Study 3 | 644 | 67.40 | 18.30 | 0.31 | 5.43 | 0.31 | 8.25 |
| Study 4 | 628 | 60.80 | 27.10 | 0.64 | 3.34 | 0.00 | 8.12 |
| Study 5 | 641 | 70.20 | 20.60 | 0.78 | 1.72 | 0.00 | 6.70 |

a Those participants who chose the option “Other” in Study 1 and Study 2 identified themselves as Arabic, Hispanic, human, Latino, Mestizo, Mexican, multiracial, Romanian, Trinidadian, or had non-specific responses.

whose self-interests were aided in the unfair decision judged the agent more moral (*M* = 4.35, *SD*

= 1.53) than did those whose self-interests were harmed in a just decision (*M* = 3.88, *SD* = 1.63), Welch’s *t*(338.7) = 2.73, *p* = .007, *d* = -0.30, 95% CI [0.13, 0.80]. In contrast, in the education condition, the effect of self-interest impact on moral character judgments was eliminated Welch’s *t*(309.6) = 1.23, *p* = 0.221, *d* = 0.14, 95% CI [-0.54, 0.12]. There were no main effects of self- interest, *F*(1, 660) = 1.38, *p* = .241, *η²*_p_ = 0.002, 95% CI [-0.38, 0.09], and education, *F*(1, 660) =

0.15, *p* = .697, *η²*_p_ = 0.000, 95% CI [-0.19, 0.28].

In Study 5, a 2 (self-interest: involved vs. not involved) × 2 (education: yes vs. no) × 3 (accountability: process vs. outcome vs. control) ANOVA revealed a significant main effect of self-interest, *F*(1, 608) = 12.96, *p* < .001, *η²*_p_ = 0.020, 95% CI [0.16, 0.56]. There were no

significant main effects of education, *F*(1, 608) = 1.40, *p* = .237, *η²*_p_ = 0.002, 95% CI [-0.08, 0.32],

and accountability, *F*(1, 607) = 2.52, *p* = .081, *η²*_p_ = 0.009, 95% CI [-0.18, 0.56], nor was there

Figure 1: Results from Study 1 after excluding those participants who incorrectly answered the two manipulation check questions in the education condition (n = 24). *Note*: Error bars are the ±1 standard error of the mean.

a significant interaction of self-interest, education, and accountability, *F*(1, 607) = 0.11, *p* = .899,

*η²*_p_ = 0.000, 95% CI [-0.71, 1.06].

1.4. *The unanimous mistake rate as a covariate*

In Study 1 and Study 2, we recognized, however, that there would be a drawback to using an ex-ante unanimous mistake rate for the purpose of best practice of data analysis. That is, the statement “Your mistake rate: 23.4%” included such a unanimous rate for all participants as their performance of the main transcript task, a task to transcribe correctly as many words as possible several best-known tongue twisters (i.e., She sells sea shells by the sea shore). This elaborate deception by commission (i.e., untrue information) was manipulated *ad hoc* in reference to a pilot

study (Miazek & Bocian, 2025), which found that participants’ mean mistake rate was a little above 20% in the same task. However, the actual mistake rates may still vary across participants (*M*_Study_ _1_

= 18.2%, *SD* = 21.6%; *M*_Study_ _2_ = 17.3%, *SD* = 19.8%). Then, some participants, if not all, may have doubts about the reliability of this rate as compared to their actual mistake rates. While it is far-fetched that any survey is perfect, the extent to which the actual rate was deceptively deviated from the unanimous one—a minor flaw or survey error—may, if any, affect construct validity of the experimental manipulation and create interpretational ambiguities.

To confine construct validity, it has been suggested that suspicion probes can be included to estimate whether any deception is likely to be perceived by participants (cf., Blackhart et al., 2012). That is, participants were asked to complete a close-ended inquiry on a 7-point scale where

-2 = “*definitely not*” and 2 = “*definitely yes*” to probe for suspicion and/or awareness of their mistake rates before being debriefed. The purpose of this inquiry was to assess whether participants were suspicious of the feedback of this unanimous mistake rate given. Thus, we further excluded these participants who were deemed definitely suspicious (*n*_Study_ _1_ = 306 and *n*_Study_ _2_ = 423) from analyses, which resulted in final samples of 382 participants (*M*_age_ = 42.1, *SD* = 13.4; 50.3% female) in Study 1 and of 544 participants (*M*_age_ = 38.6, *SD* = 11.1; 47.4% female) in Study 2. We found no differences in conclusions.

More specifically, in Study 1, a 2 (decision: unjust and self-beneficial vs. just and other- beneficial) × 2 (education: yes vs. no) ANOVA again revealed a significant interaction effect (cf., Figure 2), *F*(1, 378) = 6.22, *p* = .013, *η²* = 0.02, 95% CI [-1.075, 0.910]. Post-hoc one-

*p*

way analyses of variance show that, (1) in the non-education condition, those participants whose self-interests were involved judged the agent more moral (*M* = 4.80, *SD* = 1.43) than did those whose self-interest were noninvolved (*M* = 4.27, *SD* = 1.58), *F* = 5.78, *p* = .017, *η²*_p_ = 0.03, 95%

CI [0.094, 0.957]; and, in contrast, (2) in the education condition, those participants whose self-

interests were involved (*M* = 4.40, *SD* = 1.36) did not judge the agent significantly different from those whose self-interest were noninvolved (*M* = 4.63, *SD* = 1.49), *F* = 1.18, *p* = .279, *η²*_p_ = 0.01, 95% CI [-0.183, 0.629]. There were no main effects for decision and education, *F*(1, 378)_decision_ =

0.99, *p* = .320, *η*^2^ = 0.00, 95% CI [-0.444, 0.146]; *F*(1, 378)	= 0.04, *p* = .845, *η*^2^ = 0.00,

*p*

95% CI [-0.324, 0266].

education	*p*

Figure 2: Results from Study 1 after excluding those participants who were definitely suspicious of the mistake rate given in the main task: Moral character judgments as a function of education (no or yes). *Note*: Error bars are the ˘1 standard error of the mean.

In Study 2, a 2 (decision: unjust and self-beneficial vs. just and other-beneficial) × 2 (ac- countability: accountable vs. control) ANOVA again only revealed a significant main effect for decision, *F*(1, 539) = 7.10, *p* = .008, *η²* = 0.01, 95% CI [-0.583, -0.088]. A post-hoc unpaired Welch’s *t*-test showed that those participants whose self-interests were involved judged the agent

*p*

significantly more moral (*M* = 4.64, *SD* = 1.51) than did those whose self-interest were nonin- volved (*M* = 4.30, *SD* = 1.42), *t*(537.2) = 2.67, *p* = .008, *d* = 0.23, as seen by the visual detection (see Figure 3). There was no main effect for accountability, *F*(1, 539) = 0.03, *p* = .853, *η²* = 0.00, 95% CI [-0.271, 0.224], nor was there an interaction effect, *F*(1, 539) = 0.57, *p* = .452, *η*^2^ = 0.00,

*p*

*p*

95% CI [-0.894, 0.769].

We also performed analysis of covariance (ANCOVA) tests with participants’ actual mistake rates as a covariate. In Study 1, there was no interaction effect for decision and actual mistake rates, *F*(1, 680) = 1.89, *p* = .169, *η*2 = 0.00, nor was there an interaction effect for education and actual mistake rates, *F*(1, 680) = 1.30, *p* = .255, *η*2 = 0.00. Similar to Study 1, in Study 2, there was no interaction effect for decision and actual mistake rates, *F*(1, 957) = 3.63, *p* = .057, *η*2 = 0.01, nor was there an interaction effect for accountability and actual mistake rates, *F*(1, 957) = 0.06, *p* = .810, *η*2 = 0.00.

*p*

*p*

*p*

*p*

Further analyses also show that when the agent’s decision was *unjust and self-beneficial*, those participants whose actual mistake rates were higher than the unanimous mistake rate 23.4% judged the agent significantly more moral (*M* = 4.86, *SD* = 1.55) than did those whose actual mistake rates were lower than the unanimous mistake rate (*M* = 4.17, *SD* = 1.54), Welch’s *t*(204.4) = 4.24, *p* < .001, *d* = 0.45. In contrast, when the agent’s decision was *just and other-beneficial*, those participants whose actual mistake rates were higher than the unanimous mistake rate judged the agent not significantly differently moral (*M* = 4.23, *SD* = 1.53) than did those whose actual mistake rates were lower than the unanimous mistake rate (*M* = 4.10, *SD* = 1.51), Welch’s *t*(119.0) = 0.76, *p* = .447, *d* = 0.08. These results indicate that moral character judgments increased along with actual mistake rates when self-interest was involved, but not when self-interest was noninvolved. Additional exploratory research into this phenomenon, especially when centered on reliability of

authentic task performance, is worth examining further.

Figure 3: Effect of actual mistake rates as a covariate in the main task of Study 2 on moral character judgments: Self-interest as a determiner. *Note*: The shaded curves show the nonlinear (LOESS) regression functions with 95% confidence bands.

Taken together, no tests statistics reported in the current research rose above or fell below significance as a result of including or excluding the data from any of the samples due to those participants who were definitely suspicious of the unanimous mistake rate given in the main tasks of Study 1 and Study 2. We note that our findings are fairly robust, and thus it is unlikely that this possibly influential covariate can account for the patterns we do observe.

1.5. *Statistical analyses of exploratory variables*
     1.5.1. *Study 1*

A series of individual 2 (decision: unjust and self-beneficial vs. just and other-beneficial) × 2 (education: yes vs. no) ANOVA were conducted on these exploratory variables. The results are as follows.

**Liking toward agents.** There was a significant main effect for decision, *F*(1, 684) = 100.84,

*p* < .001, *η*2 = 0.13, 95% CI [1.006, 1.495], indicating that the mean rate of liking toward agents was significantly higher when self-interest was involved than noninvolved, *M*_involved_ = 4.43, *SD* =

*p*

1.59; *M*_noninvolved_ = 3.18, *SD* = 1.68.

**Mind of agents.** There was a significant main effect for decision, *F*(1, 684) = 6.35, *p* = .012, *η*2 = 0.01, 95% CI [0.052, 0.421], indicating that the mean rate of mind of agents was significantly higher when self-interest was involved than noninvolved, *M*_involved_ = 4.92, *SD* = 1.20; *M*_noninvolved_

*p*

= 4.68, *SD* = 1.26. There was also a significant effect for education, *F*(1, 684) = 16.64, *p* < .001, *η*2 = 0.02, 95% CI [0.199, 0.568], indicating that the mean rate of mind of agents was significantly higher in the education condition than in the non-education condition, *M*_education_ = 4.99, *SD* = 1.19; *M*_non-education_ = 4.61, *SD* = 1.28.

*p*

**Satisfaction of agents’ decisions.** There was a significant main effect for decision, *F*(1, 684)

= 216.13, *p* < .001, *η*2 = 0.24, 95% CI [1.805, 2.362], indicating that the mean rate of satisfaction of agents’ decisions was significantly higher when self-interest was involved than noninvolved, *M*_involved_ = 4.87, *SD* = 1.82; *M*_noninvolved_ = 2.79, *SD* = 1.90.

*p*

**Fairness of agents’ decisions.** There was a significant main effect for decision, *F*(1, 684) = 4.97, *p* = .026, *η*2 = 0.01, 95% CI [0.039, 0.615], indicating that the mean rate of fairness of agents’ decisions was significantly higher when self-interest was involved than noninvolved, *M*_involved_ = 3.91, *SD* = 1.88; *M*_noninvolved_ = 3.58, *SD* = 1.98.

*p*

**Intention of agents’ decisions.** There was a significant main effect for decision, *F*(1, 684) =

-21.75, *p* < .001, *η*2 = 0.03, 95% CI [-0.779, 0.000], indicating that the mean rate of intention of agents’ decisions was significantly higher when self-interest was noninvolved than involved, *M*_noninvolved_ = 5.50, *SD* = 1.53; *M*_involved_ = 4.96, *SD* = 1.55.

*p*

**Mental effort of participants.** The decision ˆ education interaction effect was significant,

*F*(1, 684) = 4.67, *p* = .031, *η*^2^ = 0.01, 95% CI [-0.621, 0.808]. A post-hoc one-way ANOVA reveals

*p*

that, in the *just and other-beneficial* decision condition, those participants who were educated devoted more mental effort (*M* = 7.37, *SD* = 1.34) than did those who were not educated (*M* = 6.96, *SD* = 1.58), *F* = 6.74, *p* = .010, *η²*_p_ = 0.02, 95% CI [0.099, 0.718].

**Recalls of the mistake rates.** The large majority of participants, 85.3% and 71.5%, declared that they remembered their own and the other passive recipient’s mistake rates, respectively.

**Purpose of the study.** We preprocessed the texts written in response to the question about the purpose of the study participants had in mind and generated a unigram word cloud (see Figure 4). **Suspicions of the mistake rate.** The majority of participants, 44.5%, declared that they were definitely suspicious of the mistake rate of 23.4% given in the main task. Figure 5 shows participant

distributions of the ratings of suspicions of the mistake rate.

**Liking as a mediator.** Past research indicated that moral evaluations are positively mediated by patients’ liking toward agents, as evidenced by judging agents morally better when they have highly attitudinal or interpersonal similarities (Bocian et al., 2018, 2022a,b) and by perceiving more responsibilities to espouse among Americans (Miller & Bersoff, 1998). To test whether liking mediates the relationship between the independent variable of decision and the measure of moral character judgments, we run a mediation analysis of PROCESS-macro Model 4 with a bootstrap method using 5,000 bootstrapped samples and 95% confidence intervals (cf., Figure 6). The results indicate that the independent variable of decision predicted the probability of (1) moral character judgments (path c), *b* = 0.80, *t*(686) = 7.35, *p* < .001, 95% CI [0.625, 0.980]; (2) moral character judgments when controlling for liking (path c’), *b* = 0.70, *t*(686) = 7.35, *p* < .001, 95% CI [-0.881, -0.510]; and (3) liking (path a), *b* = 1.25, *t*(686) = 10.04, *p* < .001, 95% CI [1.006,

1.495]. Liking significantly predicted the probability of making moral character judgments (path b), *b* = 0.64, *t*(686) = 23.60, *p* < .001, 95% CI [0.586, 0.692]. The indirect effect of decision on

moral character judgments was significant and negative (a ˆ b), *b* = 0.80, bootstrapped 95% CI

Figure 4: Inverse-document-frequency-weighted word cloud showing common single words in sizes proportional to their frequency in the response to the free-text question (on what purpose participants thought about the study) in Study 1.

[0.625, 0.980]. Because the bootstrap confidence interval did not include zero, this suggests that liking mediated the relationship between the independent variable of decision and the measure of moral character judgments.

Figure 5: Participant distributions of the ratings of suspicions of the mistake rate in response to the question, “Were you suspicious of your mistake rate of 23.4% given in the main task?”, in Study 1. *Note*. -3 = *Definitely not*, 0 = *I am not sure*, 3 = *Definitely yes*

a

Decision

Liking toward agents

< .001

*b* = 1.25, *t*(686) = 10.04, *p*

c’, *b* = 0.70, *t*(686) = 7.35, *p* < .001 c, *b* = 0.80, *t*(686) = 7.35, *p* < .001

b

*b* = 0.64, *t*(686) = 23.60, *p* < .001

Moral character judgments

Figure 6: Path diagram showing mediation analysis in Study 1. *Note*: *b*: Unstandardized regression coefficient; Path c’: Direct effect; Path c: Indirect effect.

1.5.2. *Study 2*

A series of individual 2 (decision: unjust and self-beneficial vs. just and other-beneficial) ×

2 (accountability: accountable vs. control) ANOVA were conducted on the exploratory variables.

Replicating the previous study, the results are as follows.

**Liking toward agents.** There was a significant main effect for decision, *F*(1, 962) = 180.35, *p* < .001, *η*2 = 0.16, 95% CI [1.182, 1.587], indicating that the mean rate of liking toward agents was significantly higher when self-interest was involved than noninvolved, *M*_involved_ = 4.63, *SD* =

*p*

1.63; *M*_noninvolved_ = 3.24, *SD* = 1.57.

**Mind of agents.** There was a significant main effect for decision, *F*(1, 962) = 22.11, *p* < .001, *η*2 = 0.02, 95% CI [0.218, 0.531], indicating that the mean rate of mind of agents was significantly higher when self-interest was involved than noninvolved, *M*_involved_ = 4.86, *SD* = 1.21; *M*_noninvolved_

*p*

= 4.48, *SD* = 1.27.

**Satisfaction of agents’ decisions.** There was a significant main effect for decision, *F*(1, 962)

= 347.01, *p* < .001, *η*2 = 0.27, 95% CI [1.975, 2.440], indicating that the mean rate of satisfaction of agents’ decisions was significantly higher when self-interest was involved than noninvolved, *M*_involved_ = 5.05, *SD* = 1.87; *M*_noninvolved_ = 2.85, *SD* = 1.82.

*p*

**Fairness of agents’ decisions.** There was a significant main effect for decision, *F*(1, 962) = 4.53, *p* = .034, *η*2 = 0.01, 95% CI [0.021, 0.522], indicating that the mean rate of fairness of agents’ decisions was significantly higher when self-interest was involved than noninvolved, *M*_involved_ = 4.04, *SD* = 2.01; *M*_noninvolved_ = 3.77, *SD* = 1.96.

*p*

**Intention of agents’ decisions.** There was a significant main effect for decision, *F*(1, 962) =

-17.54, *p* < .001, *η*2 = 0.02, 95% CI [-0.603, -0.218], indicating that the mean rate of intention of agents’ decisions was significantly higher when self-interest was noninvolved than involved, *M*_noninvolved_ = 5.31, *SD* = 1.49; *M*_involved_ = 4.90, *SD* = 1.55.

*p*

**Recalls of the mistake rates.** The large majority of participants, 87.7% and 74.2%, declared that they remembered their own and the other passive recipient’s mistake rates, respectively.

**Written justification.** We preprocessed the texts written in response to written justifications

about the reasons for their judgments of the agents and generated a unigram word cloud (see Figure 7).

Figure 7: Inverse-document-frequency-weighted word cloud showing common single words in sizes proportional to their frequency in the response to the free-text question (on what reasons participants justified their judgments of the agents) in Study 2.

**Suspicions of the mistake rate.** The majority of participants, 43.7%, declared that they were definitely suspicious of the mistake rate of 23.4% given in the main task. Figure 8 shows participant distributions of the ratings of suspicions of the mistake rate.

Figure 8: Participant distributions of the ratings of suspicions of the mistake rate in response to the question, “Were you suspicious of your mistake rate of 23.4% given in the main task?”, in Study 2. *Note*. -3 = *Definitely not*, 0 = *I am not sure*, 3 = *Definitely yes*

**Purpose of the study.** We preprocessed the texts written in response to the question about the purpose of the study participants had in mind and generated a unigram word cloud (see Figure 9). **Liking as a mediator.** As in Study 1, the results of mediation analysis indicate that the inde- pendent variable of decision predicted the probability of (1) moral character judgments (path c), *b* = 0.88, *t*(963) = 8.19, *p* < .001, 95% CI [-1.027, -0.732]; (2) moral character judgments when

controlling for liking (path c’), *b* = 0.67, *t*(963) = 8.19, *p* < .001, 95% CI [0.509, 0.829]; and (3)

liking (path a), *b* = 1.39, *t*(963) = 13.42, *p* < .001, 95% CI [-1.587, -1.182]. Liking significantly predicted the probability of making moral character judgments (path b), *b* = 0.63, *t*(963) = 27.03, *p* < .001, 95% CI [0.587, 0.679]. The indirect effect of decision on moral character judgments

Figure 9: Inverse-document-frequency-weighted word cloud showing common single words in sizes proportional to their frequency in the response to the free-text question (on what purpose participants thought about the study) in Study 2.

was significant and negative (a × b), *b* = 0.88, bootstrapped 95% CI [-1.027, -0.732]. Because the bootstrap confidence interval did not include zero, this suggests that liking mediated the relation- ship between the independent variable of decision and the measure of moral character judgments (cf., Figure 10).

a

Decision

Liking toward agents

< .001

*b* = 1.39, *t*(963) = 13.42, *p*

c’, *b* = 0.67, *t*(963) = 8.19, *p* < .001 c, *b* = 0.88, *t*(963) = 8.19, *p* < .001

b

*b* = 0.63, *t*(963) = 27.03, *p* < .001

Moral character judgments

Figure 10: Path diagram showing mediation analysis in Study 2. *Note*: *b*: Unstandardized regression coefficient; Path c’: Direct effect; Path c: Indirect effect.

1.5.3. *Study 3*

A series of individual 2 (self-interest: involved vs. noninvolved) × 2 (accountability: account- able vs. control) ANOVA were conducted on the exploratory variables. The results are as follows. **Liking toward agents.** There was a significant main effect for self-interest, *F*(1, 640) = 30.73,

*p* < .001, *η*^2^ = 0.05, 95% CI [0.378, 0.792], indicating that the mean rate of liking toward agents

*p*

was significantly higher when self-interest was involved than was in the control condition, *M*_involved_

= 3.63, *SD* = 1.43; *M*_control_ = 3.05, *SD* = 1.24.

**Harm of agents’ acts.** There was a significant main effect for self-interest, *F*(1, 640) = 9.91, *p*

= .024, *η*2 = 0.01, 95% CI [0.032, 0.464], indicating that the mean rate of harm of agents’ acts was significantly higher when self-interest was involved than was in the control condition, *M*_involved_ = 3.40, *SD* = 1.43; *M*_control_ = 3.15, *SD* = 1.35.

*p*

**Liking as a mediator.** As in Study 1, the results of mediation analysis indicate that self- interest failed to predict the probability of (1) moral character judgments (path c), *b* = 0.42, *t*(642)

= 0.62, *p* = .534, 95% CI [-0.570, -0.273]; and (2) moral character judgments when controlling for

liking (path c’), *b* = 0.03, *t*(642) = 0.62, *p* = .534, 95% CI [-0.138, 0.071]. However, self-interest predicted the probability of liking (path a), *b* = 0.59, *t*(642) = 5.55, *p* < .001, 95% CI [-0.792,

-0.378]. Liking significantly predicted the probability of making moral character judgments (path

b), *b* = 0.72, *t*(642) = 36.81, *p* < .001, 95% CI [0.679, 0.756]. The indirect effect of self-interest on moral character judgments was not significant and negative (a × b), *b* = 0.42, bootstrapped 95% CI [-0.570, -0.273]. Because the bootstrap confidence interval did not include zero, this suggests that liking mediated the relationship between self-interest and moral character judgments (cf., Figure 11).

a

Self- interest

Liking toward agents

< .001

*b* = 0.59, *t*(642) = 5.55, *p*

c’, *b* = 0.03, *t*(642) = 0.62, *p* = .534 c, *b* = 0.42, *t*(642) = 0.62, *p* = .534

b

*b* = 0.72, *t*(642) = 36.81, *p* < .001

Moral character judgments

Figure 11: Path diagram showing mediation analysis in Study 3. *Note*: *b*: Unstandardized regression coefficient; Path c’: Direct effect; Path c: Indirect effect.

1.5.4. *Study 4*

A series of individual 2 (self-interest: involved vs. control) × 2 (accountability: accountable - norms of justice vs. accountable - control) ANOVA were conducted on the exploratory variables. The results are as follows.

**Liking toward the agent.** There was a significant main effect for self-interest, *F*(1, 624) = 13.27, *p* < .001, *η*2 = 0.02, 95% CI [0.212, 0.710], indicating that the mean rate of liking toward the agent was significantly higher when self-interest was involved than was in the control condition, *M*_involved_ = 3.98, *SD* = 1.63; *M*_control_ = 3.52, *SD* = 1.54.

*p*

**Liking as a mediator.** As in Study 1, the results of mediation analysis indicate that self- interest failed to predict the probability of (1) moral character judgments (path c), *b* = 0.35, *t*(626)

= 0.41, *p* = .685, 95% CI [-0.544, -0.168]; and (2) moral character judgments when controlling for

liking (path c’), *b* = 0.03, *t*(626) = 0.41, *p* = .685, 95% CI [-0.182, 0.120]. However, self-interest

predicted the probability of liking (path a), *b* = 0.46, *t*(626) = 3.65, *p* = .001, 95% CI [-0.710,

-0.213]. Liking significantly predicted the probability of making moral character judgments (path b), *b* = 0.77, *t*(626) = 31.80, *p* < .001, 95% CI [0.718, 0.812]. The indirect effect of self-interest on moral character judgments was not significant and negative (a × b), *b* = 0.35, bootstrapped 95% CI [-0.544, -0.168]. Because the bootstrap confidence interval did not include zero, this suggests that liking mediated the relationship between self-interest and moral character judgments (cf., Figure 11).

1.5.5. *Study 5*

A series of individual 2 (self-interest: involved vs. not involved) × 2 (education: yes vs. no) × 3 (accountability: process vs. outcome vs. control) ANOVA were conducted on the exploratory variables. The results are as follows.

**Liking toward agents.** There was a significant main effect for self-interest, *F*(1, 628) = 18.20, *p* = .003, *η*2 = 0.01, 95% CI [0.118, 0.556], indicating that the mean rate of liking toward agents was significantly higher when self-interest was involved than not involved, *M*_involved_ = 3.61, *SD* =

*p*

1.47; *M*_not_ _involved_ = 3.27, *SD* = 1.34.

**Liking as a mediator.** As in Study 1, the results of mediation analysis indicate that self- interest predicted the probability of (1) moral character judgments (path c), *b* = 0.12, 95% CI [0.002, 0.239]; (2) moral character judgments when controlling for liking (path c’), *b* = 0.12, *t*(636) = 2.00, *p* = .046, 95% CI [0.002, 0.239]; and (3) liking (path a), *b* = 0.34, *t*(636) = 3.03,

*p* = .003, 95% CI [0.119, 0.557]. Liking significantly predicted the probability of making moral character judgments (path b), *b* = 0.73, *t*(636) = 34.12, *p* < .001, 95% CI [0.685, 0.768]. The indirect effect of self-interest on moral character judgments was significant and positive (a × b), *b* = 0.25, bootstrapped 95% CI [0.002, 0.239]. Because the bootstrap confidence interval did not

a

*b* = 0.46, *t*(626) = 3.65, *p* = .001

Self- interest

c’, *b* = 0.03, *t*(626) = 0.41, *p* = .685 c, *b* = 0.35, *t*(626) = 0.41, *p* = .685

Liking toward the agent

b

*b* = 0.77, *t*(626) = 31.80, *p* < .001

Moral character judgments

Figure 12: Path diagram showing mediation analysis in Study 4. *Note*: *b*: Unstandardized regression coefficient; Path c’: Direct effect; Path c: Indirect effect.

include zero, this suggests that liking mediated the relationship between self-interest and moral character judgments (cf., Figure 13).

Liking toward agents

a

*b* = 0.34, *t*(636) = 3.03, *p* = .003

Self- interest

c’, *b* = 0.12, *t*(636) = 2.00, *p* = .046 c, *b* = 0.25

b

*b* = 0.73, *t*(636) = 34.12, *p* < .001

Moral character judgments

Figure 13: Path diagram showing mediation analysis in Study 5. *Note*: *b*: Unstandardized regression coefficient; Path c’: Direct effect; Path c: Indirect effect.

# 2. Variables included in the surveys of the present studies on Qualtrics

2.1. *Study 1*

See Table 2.

Table 2: Variables included in the survey of Study 1 on Qualtrics.

20

Variable name	Description	Measurement	Remarks

# Main dependent variables

Moral character judg-	On a 7-point scale (1 = I strongly disagree, 7 = I strongly agree); *This*

| M1 |  | ments of agents | *decider is honest* | Item 1 of 5 |
| --- | --- | --- | --- | --- |
| M2 |  | Ibid. | Ibid.; *This decider is reliable* | Item 2 of 5 |
| M3 |  | Ibid. | Ibid.; *This is a trustworthy decider* | Item 3 of 5 |
| M4 |  | Ibid. | Ibid.; *This decider is moral* | Item 4 of 5 |
| M5 |  | Ibid. | Ibid.; *This decider is just* | Item 5 of 5 |
| **Other ploratory dependent**  **variables** | **ex-** |  |  |  |
| future |  | Liking toward agents | Ibid.; *I would like to interact with this decider in the future* | Item 1 of 2 |
| like |  | Ibid. | Ibid.; *I like this decider* | Item 2 of 2 |
| a1 |  | Mind of agents | Ibid.; *This decider can communicate with others* | Item 1 of 6 |

Table 2: Variables included in the survey of Study 1 on Qualtrics. (continued)

Variable name	Description	Measurement	Remarks

a2	Ibid.	Ibid.; *This decider is capable of thinking*	Item 2 of 6

a3	Ibid.	Ibid.; *This decider plans his/her actions*	Item 3 of 6

e1	Ibid.	Ibid.; *This decider is sensitive to pain*	Item 4 of 6

e2	Ibid.	Ibid.; *This decider can experience happiness*	Item 5 of 6

e3	Ibid.	Ibid.; *This decider can experience fear*	Item 6 of 6

Satisfiy

Satisfaction of agents’ decisions

On a 7-point scale (1 = Not at all, 7 = Extremely); *How satisfied are you with the outcome of the decision?*

fair_decision

Fairness of agents’ de- cisions

Ibid.; *How fair was the decision?*

intentional

Intention of agents’ de- cisions

Ibid.; *How intentional was the decision?*

21

Q216

Mental effort of partici- pants

On a 9-point scale (1 = Very, very low mental effort, 9 = Very, very high mental effort); *Please indicate on the scale below how much mental effort you have invested in answering all questions about the decider.*

Paas (1992)

Table 2: Variables included in the survey of Study 1 on Qualtrics. (continued)

Variable name	Description	Measurement	Remarks

# Independent vari- ables

Education

Education	manipula- tion

Participants were randomly assigned to the education and non- education conditions

Self_interest	Decision manipulation

# Other variables / covariates

Participants were randomly assigned to the unjust and self-beneficial and just and other-beneficial conditions

*Please indicate if the following statement is TRUE or FALSE. The self-*

In the *Education*

Q221

Manipulation	check question 1

*interest bias is a tendency to judge people as more moral when their action benefits us, albeit violates a norm, such as fairness, and less moral if the same action benefits someone else.*

and	*Unjust	but self-beneficial* condition

22

Table 2: Variables included in the survey of Study 1 on Qualtrics. (continued)

Variable name	Description	Measurement	Remarks

*Please indicate if the following statement is TRUE or FALSE. To*

Q224

Manipulation	check question 2

*avoid the self-interest bias, our judgments on people should depend on whether their actions are against any norm, such as fairness, rather than our personal interests.*

Ibid.

*Please indicate if the following statement is TRUE or FALSE. The self-*	In the *Education*

Q225

Manipulation	check question 3

*interest bias is a tendency to judge people as more moral when their action benefits us, albeit violates a norm, such as fairness, and less moral if the same action benefits someone else.*

and	*Just	but other-beneficial* condition

*Please indicate if the following statement is TRUE or FALSE. To*

Q227

Manipulation	check question 4

*avoid the self-interest bias, our judgments on people should depend on whether their actions are against any norm, such as fairness, rather than our personal interests.*

Ibid.

23

Finished

Whether	participants

finished the survey

0 = Not finished, 1 = finished

Table 2: Variables included in the survey of Study 1 on Qualtrics. (continued)

Variable name	Description	Measurement	Remarks

Att_check

Attention check ques- tion

On a 7-point scale (1 = I strongly disagree, 7 = I strongly agree); *If you are reading this, pick six*

Recalls of the partic-

recall_self

ipants’	own	mistake rates

A yes-no check question; *Do you remember your mistake rate?*

recall_self_open	Ibid.	An open-ended question; *What was your mistake rate?*

Recalls of the mistake

recall_other

rates of the others with whom participants were matched

A yes-no check question; *Do you remember the other participant’s mis- take rate?*

recall_other_open	Ibid.

An open-ended question; *What was the other participant’s mistake rate?*

24

task

Participants’	engage-

ment

Whether participants devoted a low level of engagement to the main

transcript task, as evidenced by transcribing less than 5 words

Table 2: Variables included in the survey of Study 1 on Qualtrics. (continued)

Variable name	Description	Measurement	Remarks

Gender	Participants’ gender	1 = Female, 2 = Male, 3 = Other/prefer not to say Age	Participants’ age

Race

Participants’ ethnic cat- egories

1 = Caucasian, 2 = African American, 3 = American Indian or Alaska Native, 4 = Asian, 5 = Native Hawaiian or Pacific Islander, 6 = Other

Race_6_TEXT	Ibid.

An open-ended text field is provided when participants chose “Other” in the above question

Purpose	Purpose of the study

An open-ended question; *Can you guess what was the real purpose of the study? Enter your observations below.*

Q205

Suspicions of the mis- take rates

On a 7-point scale (1 = Definitely not, 7 = Definitely yes); *Were you suspicious of your mistake rate of 23.4% given in the main task?*

25

Table 2: Variables included in the survey of Study 1 on Qualtrics. (continued)

Variable name	Description	Measurement	Remarks

total_mistakes

Participants’	actual mistakes

Number of words that participants incorrectly transcribed in the main transcript task

total_words_typed

Total words that partic- ipants typed

Number of words that participants typed in the main transcript task

26

2.2. *Study 2*

Different from those variables included in the survey of Study 1, in Study 2, (1) the variables Q221, Q224, Q225, Q227, and Education were not included; and (2) the variables Accountability and Q243 were included (see Table 3). The rest were the same as those included in the survey of

Study 1.

Table 3: Variables exclusively included in the survey of Study 2 on Qualtrics.

Variable name	Description	Measurement

# Independent variables

Accountability

# Other variables / covariates

Q243

Accountability manipulation

Written justifica- tion

Participants were randomly as- signed to the accountable and con- trol conditions

An open-ended question; *Please justify the reasons behind your judgments of the Decider.*

2.3. *Study 3*

See Table 4.

2.4. *Study 4*

Different from those variables included in the survey of Study 3, in Study 4, (1) the variables Mental_Effort and Manipulation_Check were not included; and (2) the variables Check_Accoun_Norm

27

Table 4: Variables included in the survey of Study 3 on Qualtrics.

Variable name	Description	Measurement	Remarks

# Main dependent vari- ables

Moral	character	On a 7-point scale (1 = Strongly dis-

Moral_Class_1

judgments	of agents

agree, 7 = Strongly agree); *Kate is fair*

Item 1 of 5 in the Vignette-Class

Moral_Political_1	Ibid.	Ibid.	Item 1 of 5 in the Vignette-Politics

28

Moral_Organization_1	Ibid.	Ibid.	Item 1 of 5 in the Vignette-Organization

Moral_Soft_1	Ibid.	Ibid.	Item 1 of 5 in the Vignette-Soft Moral_Class_2	Ibid.	Ibid.; *Kate is moral*	Item 2 of 5 in the Vignette-Class Moral_Political_2	Ibid.	Ibid.	Item 2 of 5 in the Vignette-Politics

Moral_Organization_2	Ibid.	Ibid.	Item 2 of 5 in the Vignette-Organization

Moral_Soft_2	Ibid.	Ibid.	Item 2 of 5 in the Vignette-Soft Moral_Class_3	Ibid.	Ibid.; *Kate is trustworthy*	Item 3 of 5 in the Vignette-Class Moral_Political_3	Ibid.	Ibid.	Item 3 of 5 in the Vignette-Politics

Table 4: Variables included in the survey of Study 3 on Qualtrics. (continued)

29

| Variable name |  | Description | Measurement | Remarks |
| --- | --- | --- | --- | --- |
| Moral_Organization_3 |  | Ibid. | Ibid. | Item 3 of 5 in the Vignette-Organization |
| Moral_Soft_3 |  | Ibid. | Ibid. | Item 3 of 5 in the Vignette-Soft |
| Moral_Class_4 |  | Ibid. | Ibid.; *Kate is reliable* | Item 4 of 5 in the Vignette-Class |
| Moral_Political_4 |  | Ibid. | Ibid. | Item 4 of 5 in the Vignette-Politics |
| Moral_Organization_4 |  | Ibid. | Ibid. | Item 4 of 5 in the Vignette-Organization |
| Moral_Soft_4 |  | Ibid. | Ibid. | Item 4 of 5 in the Vignette-Soft |
| Moral_Class_5 |  | Ibid. | Ibid.; *Kate is honest* | Item 5 of 5 in the Vignette-Class |
| Moral_Political_5 |  | Ibid. | Ibid. | Item 5 of 5 in the Vignette-Politics |
| Moral_Organization_5 |  | Ibid. | Ibid. | Item 5 of 5 in the Vignette-Organization |
| Moral_Soft_5 |  | Ibid. | Ibid. | Item 5 of 5 in the Vignette-Soft |
| **Other exploratory** | **de-** |  |  |  |
| **pendent variables** |  |  |  |  |
| Liking_Class_1 |  | Liking toward agents | On a 7-point scale (1 = Strongly dis-  agree, 7 = Strongly agree); Ibid.; *I like Kate* | Item 1 of 2 in the Vignette-Class |

Table 4: Variables included in the survey of Study 3 on Qualtrics. (continued)

Variable name	Description	Measurement	Remarks

Liking_Political_1	Ibid.	Ibid.

Item 1 of 2 in the Vignette-Politics

Liking_Organization_1	Ibid.	Ibid.

Item 1 of 2 in the Vignette-Organization

Liking_Soft_1	Ibid.	Ibid.

Item 1 of 2 in the Vignette-Soft

Liking_Class_2	Ibid.	Ibid.; *I would like to meet Kate in the future*

Item 2 of 2 in the Vignette-Class

Liking_Political_2	Ibid.	Ibid.

Item 2 of 2 in the Vignette-Politics

Liking_Organization_2	Ibid.	Ibid.

Item 2 of 2 in the Vignette-Organization

30

Table 4: Variables included in the survey of Study 3 on Qualtrics. (continued)

Variable name	Description	Measurement	Remarks

Liking_Soft_2	Ibid.	Ibid.

Item 2 of 2 in the Vignette-Soft

On a 7-point scale (1 = Completely Harmful,

Harmful_Class	Harm of agents’ acts

7 = Completely Unharmful); *How harmful was Kate’s decision?*

In the Vignette-Class

Harmful_Political	Ibid.	Ibid.	In the Vignette-Politics

Harmful_Organization	Ibid.	Ibid.

In	the	Vignette- Organization

Harmful_Soft	Ibid.	Ibid.	In the Vignette-Soft

On a 9-point scale (1 = Very, very low mental

31

Mental_Effort

Mental effort of partici- pants

effort, 9 = Very, very high mental effort); *Please indicate how much mental effort you have in- vested in answering all the questions above.*

Paas (1992)

Table 4: Variables included in the survey of Study 3 on Qualtrics. (continued)

Variable name	Description	Measurement	Remarks

# Independent variables

Accountability

Accountability manipu- lation

Participants were randomly assigned to the ac- countable and control conditions

Self_interest

# Other variables / covari- ates

Finished

Self-interest manipula- tion

Whether	participants finished the survey

Participants were randomly assigned to the in- volved and control conditions

0 = Not finished, 1 = finished

Class_Check_Question

Vignette	comprehen- sion check question

A yes-no question; *Did Kate copy the answers from her friend?*

In the Vignette-Class

Political_Check_Ques	Ibid.	Ibid.; *Did Kate delete the citizen’s fine?*	In the Vignette-Politics

32

Table 4: Variables included in the survey of Study 3 on Qualtrics. (continued)

Variable name	Description	Measurement	Remarks

Organization_Check_Q	Ibid.	Ibid.; *Did Kate offer a promotion to Tom?*

In	the	Vignette- Organization

Soft_Check_Question	Ibid.	Ibid.; *Did Kate download the costly software?*	In the Vignette-Soft

Manipulation_Check

Manipulation	check question

On a 7-point scale (1 = Definitely Not, 7 = Def- initely Yes); *Do you expect to explain the rea- sons for your judgments to an experimenter?*

Gender	Participants’ gender

1 = Female, 2 = Male, 3 = Other/prefer not to say

Age	Participants’ age

1 = White or Caucasian, 2 = Black or African

Race

Participants’ ethnic cat- egories

American, 3 = American Indian/Native Amer- ican or Alaska Native, 4 = Asian, 5 = Native Hawaiian or Other Pacific Islander, 6 = Other, 7

= Prefer not to say

33

and Check_Accoun_Control were included (see Table 5). The rest were the same as those included in the survey of Study 3.

Table 5: Variables exclusively included in the survey of Study 4 on Qualtrics.

Variable name	Description	Measurement	Remarks

# Other variables / covariates

On a 7-point scale (1 = Def-

Check_Accoun_Norm

Manipulation check question 1

initely Not, 7 = Definitely Yes); *Do you expect to ex- plain the reasons for your judgments to an experimenter according to social and eth- ical norms such as norms of justice?*

In the ac- countable to	norms

of	justice condition

Ibid.;	*Do	you	expect	to*	In	the

Check_Accoun_Control

Manipulation check question 2

*explain the reasons for your judgments to an experi- menter?*

only	ac- countable condition

2.5. *Study 5*

Different from those variables included in the survey of Study 3, in Study 5, (1) the variables Mental_Effort was not included; and (2) the variables Education, Accountability, Check_Process_Bias_1, and Check_Process_Bias_2 were included (see Table 6). The rest were the same as those included

in the survey of Study 3.

Table 6: Variables exclusively included in the survey of Study 5 on Qualtrics.

Variable name	Description	Measurement	Remarks

# Independent variables

Participants were randomly

Education

Education manip- ulation

assigned to the education - yes and education - no condi- tions

Accountability

# Other variables / covariates

Check_Process_Bias_1

Accountability manipulation

Education ma- nipulation check question 1

Participants were randomly assigned to the process, out- come, and control conditions

*Please indicate if the follow- ing statement is TRUE or FALSE. The self-interest bias is a tendency to judge peo- ple as more moral when their action benefits us, albeit vio- lates a norm, such as fairness, and less moral if the same ac- tion benefits someone else.*

In the *Edu- cation - yes* condition

Table 6: Variables exclusively included in the survey of Study 5 on Qualtrics. (continued)

| Variable name | Description |  | Measurement | Remarks |
| --- | --- | --- | --- | --- |
|  |  |  | *Please indicate if the follow-* |  |
|  |  |  | *ing statement is TRUE or* |  |
|  |  |  | *FALSE. To avoid the self-* |  |
|  | Education | ma- | *interest bias, our judgments* |  |
| Check_Process_Bias_2 | nipulation | check | *on people should depend on* | Ibid. |
|  | question 2 |  | *whether their actions are* |  |
|  |  |  | *against any norm, such as* |  |
|  |  |  | *fairness, rather than our per-* |  |
|  |  |  | *sonal interests.* |  |

# Bibliography

Blackhart, G. C., Brown, K. E., Clark, T., Pierce, D. L., & Shell, K. (2012). Assessing the adequacy of postexperimen- tal inquiries in deception research and the factors that promote participant honesty. *Behavior Research Methods*, *44*, 24–40. doi:[10.3758/s13428-011-0132-6](http://dx.doi.org/10.3758/s13428-011-0132-6).

Bocian, K., Baryla, W., Kulesza, W. M., Schnall, S., & Wojciszke, B. (2018). The mere liking effect: Attitudinal

influences on attributions of moral character. *Journal of Experimental Social Psychology*, *79*, 9–20. doi:[10.1016/](http://dx.doi.org/10.1016/j.jesp.2018.06.007) [j.jesp.2018.06.007](http://dx.doi.org/10.1016/j.jesp.2018.06.007).

Bocian, K., Baryla, W., & Wojciszke, B. (2022a). Moderators of the liking bias in judgments of moral character.

*Personality and Social Psychology Bulletin*, *48*, 596–605. doi:[10.1177/01461672211013272](http://dx.doi.org/10.1177/01461672211013272).

Bocian, K., Szarek, K. M., Miazek, K., Baryla, W., & Wojciszke, B. (2022b). The boundary conditions of the liking bias in moral character judgments. *Scientific Reports*, *12*, Nr. 17217. doi:[10.1038/s41598-022-22147-7](http://dx.doi.org/10.1038/s41598-022-22147-7).

Miazek, K., & Bocian, K. (2025). When AI is fairer than humans: The role of egocentrism in moral and fairness

judgments of AI and human decisions. doi:[10.1016/j.chbr.2025.100719](http://dx.doi.org/10.1016/j.chbr.2025.100719).

Miller, J. G., & Bersoff, D. M. (1998). The role of liking in perceptions of the moral responsibility to help: A culture

perspective. *Journal of Experimental Social Psychology*, *34*, 443–469. doi:[10.1006/jesp.1998.1359](http://dx.doi.org/10.1006/jesp.1998.1359).

Paas, F. (1992). Training strategies for attaining transfer of problem-solving skill in statistics: A cognitive-load approach. *Journal of Educational Psychology*, *84*, 429–434. doi:[10.1037/0022-0663.84.4.429](http://dx.doi.org/10.1037/0022-0663.84.4.429).
